# Spreading depolarization and angiographic spasm are separate mediators of delayed infarcts

**DOI:** 10.1093/braincomms/fcad080

**Published:** 2023-03-22

**Authors:** Viktor Horst, Vasilis Kola, Coline L Lemale, Sebastian Major, Maren K L Winkler, Nils Hecht, Edgar Santos, Johannes Platz, Oliver W Sakowitz, Hartmut Vatter, Christian Dohmen, Michael Scheel, Peter Vajkoczy, Jed A Hartings, Johannes Woitzik, Peter Martus, Jens P Dreier

**Affiliations:** Centre for Stroke Research Berlin, Charité—Universitätsmedizin Berlin, Corporate Member of Freie Universität Berlin, Humboldt-Universität zu Berlin, and Berlin Institute of Health, Berlin, Germany; Centre for Stroke Research Berlin, Charité—Universitätsmedizin Berlin, Corporate Member of Freie Universität Berlin, Humboldt-Universität zu Berlin, and Berlin Institute of Health, Berlin, Germany; Centre for Stroke Research Berlin, Charité—Universitätsmedizin Berlin, Corporate Member of Freie Universität Berlin, Humboldt-Universität zu Berlin, and Berlin Institute of Health, Berlin, Germany; Department of Experimental Neurology, Charité—Universitätsmedizin Berlin, Corporate Member of Freie Universität Berlin, Humboldt-Universität zu Berlin, and Berlin Institute of Health, Berlin, Germany; Centre for Stroke Research Berlin, Charité—Universitätsmedizin Berlin, Corporate Member of Freie Universität Berlin, Humboldt-Universität zu Berlin, and Berlin Institute of Health, Berlin, Germany; Department of Experimental Neurology, Charité—Universitätsmedizin Berlin, Corporate Member of Freie Universität Berlin, Humboldt-Universität zu Berlin, and Berlin Institute of Health, Berlin, Germany; Department of Neurology, Charité—Universitätsmedizin Berlin, Corporate Member of Freie Universität Berlin, Humboldt-Universität zu Berlin, and Berlin Institute of Health, Berlin, Germany; Centre for Stroke Research Berlin, Charité—Universitätsmedizin Berlin, Corporate Member of Freie Universität Berlin, Humboldt-Universität zu Berlin, and Berlin Institute of Health, Berlin, Germany; Robert Koch Institute, Berlin, Germany; Centre for Stroke Research Berlin, Charité—Universitätsmedizin Berlin, Corporate Member of Freie Universität Berlin, Humboldt-Universität zu Berlin, and Berlin Institute of Health, Berlin, Germany; Department of Neurosurgery, Charité—Universitätsmedizin Berlin, Corporate Member of Freie Universität Berlin, Humboldt-Universität zu Berlin, and Berlin Institute of Health, Berlin, Germany; Department of Neurosurgery, Heidelberg University Hospital, Ruprecht-Karls-University Heidelberg, Heidelberg, Germany; Department of Neurosurgery, Herz-Neuro-Zentrum Bodensee, Kreuzlingen, Switzerland; Department of Neurosurgery, Heidelberg University Hospital, Ruprecht-Karls-University Heidelberg, Heidelberg, Germany; Department of Neurosurgery, University Hospital and Friedrich-Wilhelms-University Bonn, Bonn, Germany; Department for Neurology and Neurological Intensive Care Medicine, LVR-Klinik Bonn, Bonn, Germany; Department of Neuroradiology, Charité—Universitätsmedizin Berlin, Corporate Member of Freie Universität Berlin, Humboldt-Universität zu Berlin, and Berlin Institute of Health, Berlin, Germany; Centre for Stroke Research Berlin, Charité—Universitätsmedizin Berlin, Corporate Member of Freie Universität Berlin, Humboldt-Universität zu Berlin, and Berlin Institute of Health, Berlin, Germany; Department of Neurosurgery, Charité—Universitätsmedizin Berlin, Corporate Member of Freie Universität Berlin, Humboldt-Universität zu Berlin, and Berlin Institute of Health, Berlin, Germany; Department of Neurosurgery, University of Cincinnati College of Medicine, Cincinnati, OH, USA; Centre for Stroke Research Berlin, Charité—Universitätsmedizin Berlin, Corporate Member of Freie Universität Berlin, Humboldt-Universität zu Berlin, and Berlin Institute of Health, Berlin, Germany; Department of Neurosurgery, Evangelisches Krankenhaus Oldenburg, University of Oldenburg, Oldenburg, Germany; Institute for Clinical Epidemiology and Applied Biometry, University of Tübingen, Tübingen, Germany; Centre for Stroke Research Berlin, Charité—Universitätsmedizin Berlin, Corporate Member of Freie Universität Berlin, Humboldt-Universität zu Berlin, and Berlin Institute of Health, Berlin, Germany; Department of Experimental Neurology, Charité—Universitätsmedizin Berlin, Corporate Member of Freie Universität Berlin, Humboldt-Universität zu Berlin, and Berlin Institute of Health, Berlin, Germany; Department of Neurology, Charité—Universitätsmedizin Berlin, Corporate Member of Freie Universität Berlin, Humboldt-Universität zu Berlin, and Berlin Institute of Health, Berlin, Germany; Bernstein Centre for Computational Neuroscience Berlin, Berlin, Germany; Einstein Centre for Neurosciences Berlin, Berlin, Germany

**Keywords:** cytotoxic oedema, spreading depolarization, spreading ischaemia, subarachnoid haemorrhage, vasospasm

## Abstract

In DISCHARGE-1, a recent Phase III diagnostic trial in aneurysmal subarachnoid haemorrhage patients, spreading depolarization variables were found to be an independent real-time biomarker of delayed cerebral ischaemia. We here investigated based on prospectively collected data from DISCHARGE-1 whether delayed infarcts in the anterior, middle, or posterior cerebral artery territories correlate with (i) extravascular blood volumes; (ii) predefined spreading depolarization variables, or proximal vasospasm assessed by either (iii) digital subtraction angiography or (iv) transcranial Doppler-sonography; and whether spreading depolarizations and/or vasospasm are mediators between extravascular blood and delayed infarcts. Relationships between variable groups were analysed using Spearman correlations in 136 patients. Thereafter, principal component analyses were performed for each variable group. Obtained components were included in path models with *a priori* defined structure. In the first path model, we only included spreading depolarization variables, as our primary interest was to investigate spreading depolarizations. Standardised path coefficients were 0.22 for the path from extravascular blood_component_ to depolarization_component_ (*P* = 0.010); and 0.44 for the path from depolarization_component_ to the first principal component of delayed infarct volume (*P* < 0.001); but only 0.07 for the direct path from blood_component_ to delayed infarct_component_ (*P* = 0.36). Thus, the role of spreading depolarizations as a mediator between blood and delayed infarcts was confirmed. In the principal component analysis of extravascular blood volume, intraventricular haemorrhage was not represented in the first component. Therefore, based on the correlation analyses, we also constructed another path model with blood_component_ without intraventricular haemorrhage as first and intraventricular haemorrhage as second extrinsic variable. We found two paths, one from (subarachnoid) blood_component_ to delayed infarct_component_ with depolarization_component_ as mediator (path coefficients from blood_component_ to depolarization_component_ = 0.23, *P* = 0.03; path coefficients from depolarization_component_ to delayed infarct_component_ = 0.29, *P* = 0.002), and one from intraventricular haemorrhage to delayed infarct_component_ with angiographic vasospasm_component_ as mediator variable (path coefficients from intraventricular haemorrhage to vasospasm_component_ = 0.24, *P* = 0.03; path coefficients from vasospasm_component_ to delayed infarct_component_ = 0.35, *P* < 0.001). Human autopsy studies shaped the hypothesis that blood clots on the cortex surface suffice to cause delayed infarcts beneath the clots. Experimentally, clot-released factors induce cortical spreading depolarizations that trigger (i) neuronal cytotoxic oedema and (ii) spreading ischaemia. The statistical mediator role of spreading depolarization variables between subarachnoid blood volume and delayed infarct volume supports this pathogenetic concept. We did not find that angiographic vasospasm triggers spreading depolarizations, but angiographic vasospasm contributed to delayed infarct volume. This could possibly result from enhancement of spreading depolarization-induced spreading ischaemia by reduced upstream blood supply.

## Teaser

Horst *et al*. report that spreading depolarization variables are a statistical mediator between subarachnoid blood volume in initial computed tomography and delayed infarct volume in longitudinal neuroimaging after subarachnoid haemorrhage, whereas angiographic vasospasm is a mediator between intraventricular haemorrhage and delayed infarct volume and that the two do not correlate

## Graphical Abstract

**Graphical Abstract:**
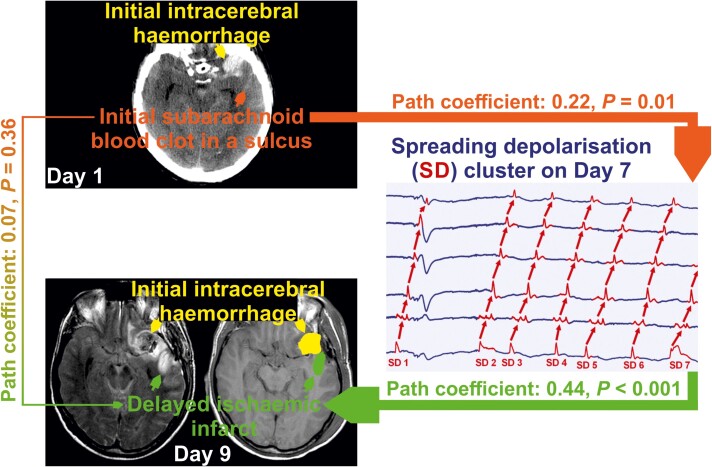

## Introduction

Subarachnoid haemorrhage (SAH) is the second most common type of haemorrhagic stroke.^1,2^ In 85%, SAH is caused by the rupture of an aneurysm. Although SAH accounts for only ∼3% of all strokes and ∼5% of deaths from stroke, the relative youth of the affected individuals means that it is responsible for a quarter of all stroke-related years of potential life lost before age 65.^3^ In Depolarisations in ISCHaemia after subARachnoid haemorrhaGE-1 (DISCHARGE-1), a recent prospective, observational, multicentre, cohort, Phase III diagnostic trial of 180 patients with severe aneurysmal SAH (aSAH), the strongest predictor of long-term outcome was total focal brain damage detected by neuroimaging two weeks after the initial haemorrhage.^4^ Most prominent aetiologies of focal brain damage associated with aSAH are intracerebral haemorrhage (ICH), and infarction due to either early (ECI), or delayed cerebral ischaemia (DCI). DISCHARGE-1 found that the average patient admitted to the neurocritical care unit after aneurysm treatment had already lost 46 ± 73 ml (mean ± standard deviation) of brain tissue due to ICH and ECI and lost an additional 36 ± 80 ml (44% of the total focal brain damage) over the next two weeks because of delayed ischaemic infarcts. This tissue could be saved if we knew effective treatments, because DCI is a potentially modifiable aetiology of focal brain damage during neurocritical care, as it allows treatment with a neuroprotective intervention before the potential insult or soon after. The risk of DCI is particularly high after severe aSAH. Thus, delayed infarct volume in DISCHARGE-1 was significantly higher in deeply comatose patients than in patients who were at least transiently clinically assessable (48 ± 92 ml versus 23 ± 74 ml, *P* < 0.001).^4^ Severe cases require mechanical ventilation and sedation more often, which limits neurological assessment. Therefore, in the high-risk population, it is particularly difficult to identify and treat those patients who suffer from the complication. However, neurosurgical procedures are indicated early after aSAH, allowing implantation of invasive probes. This enables recording of the entire period of ischaemic stroke development, early treatment stratification according to changes in diagnostic summary measures recorded by neuromonitoring devices in real time and then re-assessment of these measures after neuroprotective interventions.^5^ In awake patients, neurologic examination might be the strongest DCI predictor.^6^ However, particularly in comatose or sleeping patients, the results of DISCHARGE-1 suggest that spreading depolarization (SD) variables are currently the most promising DCI predictor.^4^

SD is a phenomenon of the brain grey matter. Using subdural electrocorticography (ECoG), it is observed as a large negative direct current (DC) shift which spreads between adjacent recording sites (frequency band: < 0.05 Hz). SD is characterised by abrupt, near-complete breakdown of the transmembrane neuronal ion gradients with entropy increase, release of 90% of Gibbs free energy normally contained in the ion gradients, neuronal water uptake, soma swelling, dendritic beading, and MRI diffusion restriction.^7-9^ Collectively, SD is the prime process that initiates and maintains neuronal cytotoxic oedema in grey matter.^9,10^ This means that SD initiates toxic changes that eventually lead to neuronal death, but is not a marker of death *per se*, as it is reversible—up to a point—with restoration of the physiological state of low entropy by Na^+^/K ^+^ -ATPase (NaKA) activation.^11^ The most important NaKA activators in this context are the extreme increases in cytoplasmic Na^+^ and extracellular K^+^ concentration, which are nowhere near as high in any other grey matter pathological phenomenon as in SD.^11-15^ However, if NaKAs cannot be sufficiently activated, e.g. due to enzyme inhibition or as a result of ATP deficiency, the neurons die, which is indicated by the transition to a negative ultraslow potential (NUP) in ECoG and a persistent diffusion restriction in MRI.^8,16-18^

Importantly, SDs induce tone alterations in resistance vessels, causing either predominant hyperperfusion followed by a mild oligaemia (physiological haemodynamic response) in healthy tissue^19,20^; or severe and prolonged initial hypoperfusion (inverse haemodynamic response = spreading ischaemia) where the neurovascular unit is severely disturbed.^5,21,22^ SD in naive tissue associated with a normal haemodynamic response does not cause neuronal damage.^23^ However, SD-induced spreading ischaemia can lead to infarction even in brain tissue that was not yet ischaemic at the onset of SD.^24^ This is because spreading ischaemia-induced ATP deficiency keeps the neurons in the SD/cytotoxic oedema state, the SD/cytotoxic oedema state maintains vasoconstriction and the vasoconstriction restricts the substrate supply for ATP production.^5^ If this vicious circle is not interrupted, it eventually leads to ischaemic necrosis.^5,21^ Spreading ischaemia is thus distinguished from primary ischaemia, such as occurs in the setting of embolic or thrombotic occlusion of a major cerebral artery or cardiocirculatory arrest. Whereas in the case of spreading ischaemia, SD occurs first and is followed by ischaemia with a latency of several seconds, and both SD and ischaemia propagate in the tissue,^5,21^ in the case of severe primary ischaemia, ischaemia occurs first, followed by SD with a substantial latency of ∼1–5 minutes, and only SD, but not ischaemia, propagates in the tissue.^16^ Nevertheless, the process of spreading ischaemia can also build up on incomplete primary ischaemia.^25-29^ If primary ischaemia of the cortex does not lead to at least one SD, infarction does not occur.^16,30-32^ If primary ischaemia leads to SD but timely reperfusion occurs, no lesion develops either.^16,33^

The main principles of the SD process known from animal experiments could be verified in experiments with human brain slices.^34-40^ The entire SD continuum from short duration, to intermediate duration, to terminal waves has now been demonstrated in aSAH patients.^4,41,42^ After aSAH, SDs have been recorded in association with (i) migraine aura^43^; (ii) transitory ischaemic attacks^4^; (iii) status epilepticus^36,44,45^; (iv) vasogenic oedema development without infarction^4^; (v) ICH^46^; (vi) early ischaemic infarcts^4,47,48^; (vii) delayed ischaemic infarcts^4,22,49^; (viii) brain death development^4,50,51^ and (ix) dying from cardiocirculatory arrest.^4,52^ The broad range of conditions under which SD has been detected in patients using ECoG closely matches the wide range of conditions under which cytotoxic oedema is detected using neuroimaging. Importantly, however, this does not mean that every SD has a correlate on clinical MRI, as SD/neuronal cytotoxic oedema is usually initially reversible and once regressed is no longer detectable on neuroimaging.^53,54^ In addition, SD-induced spreading ischaemia and transition from clustered SDs to NUP were demonstrated in a small population of aSAH patients in whom optoelectrodes for laser-Doppler flowmetry and ECoG were located directly over newly developing delayed infarcts proven by longitudinal neuroimaging.^4,16,17^

SD is associated with different changes in spontaneous brain activity in the alternating current (AC) band of the ECoG (>0.5 Hz). These are non-spreading activity depression, spreading activity depression and epileptiform activity.^11,36,52^ The same SD wave may be associated with different activity changes and different haemodynamic responses in adjacent brain regions. In DISCHARGE-1, for each recording day of each patient, we determined (i) the total (cumulative) SD-induced depression duration (TDDD) and (ii) the number of SDs as the most important ECoG variables. While the *a priori* defined 60-min cut-off of TDDD indicated a reversible delayed neurological deficit, only a 180-min cut-off indicated new infarction with >0.60 sensitivity and >0.80 specificity.^4^ On this basis, it was recommended that rescue treatment be initiated at the 60-min cut-off rather than at the 180-min cut-off if progression of injury to infarction is to be prevented. Overall, SD variables were included in each multiple regression model for early, delayed and total brain damage, 7-month outcome, and death, suggesting that they are an independent biomarker of progressive brain injury.^4^

Traditional pathology literature describes delayed infarcts after aSAH as focal anaemic necroses, suggesting arterial/arteriolar spasm as underlying aetiology and ruling out mechanisms such as thrombotic occlusion, endothelial swelling, or venous compression.^55^ SD-induced vasocontraction, the cause of spreading ischaemia, is in fact the most extreme form of vasospasm in the brain currently known.^5,21,22,24^ In addition, two slowly evolving forms of vasospasm emerge after aSAH: angiographic (proximal) vasospasm and chronic constriction of distal arteries/arterioles.^56-58^ In the autopsy studies, the predominant lesion pattern consisted of widespread infarcts in the cerebral cortex.^55,59-63^ Seventy to 80% of patients showed such cortical infarcts in the large autopsy series.^55,59^ In particular, Stoltenburg-Didinger and Schwarz^55^ noted that these lesions typically develop beneath subarachnoid clots. In animal experiments, haemolysis products in the subarachnoid space without the simultaneous presence of proximal vasospam are sufficient to cause SD, SD-induced spreading ischaemia and cortical infarction.^13,21,24,42,64,65^ However, upstream restriction of regional cerebral blood flow (rCBF), if severe enough, can also trigger SDs^66-69^ and shift the normal, predominantly hyperaemic response to SD towards an inverse ischaemic response.^25-27,70,71^ Accordingly, there are two alternative hypotheses for the development of delayed SDs after aSAH: (i) blood degradation products around the basal conductive arteries trigger angiographic vasospasm, which acts as a mediator of SDs through a mismatch between supply and demand; or (ii) blood degradation products located on the cortex trigger SDs directly in the underlying cortex through other mechanisms, including neuronal, astrocytic and microvascular disruption and/or local inflammation. To test these two hypotheses, we here investigated based on prospectively collected data from DISCHARGE-1 whether delayed infarcts in the anterior (ACA), middle (MCA) or posterior cerebral artery (PCA) territories ipsilateral to the subdural electrodes correlate with (i) extravascular blood volumes in different compartments; (ii) predefined SD variables, or proximal vasospasm assessed by either (iii) digital subtraction angiography (DSA) or (iv) transcranial Doppler-sonography (TCD); and whether proximal vasospasm and/or SD variables are mediators between extravascular blood volumes and delayed infarcts.

## Materials and methods

### Study design and protocol

This study was designed and performed as a substudy of the Depolarisations in ISCHaemia after subARachnoid haemorrhaGE-1 (DISCHARGE-1) trial.^4^ As reported previously, patients with aSAH were screened for study inclusion and were consecutively enrolled in DISCHARGE-1 at six university-hospitals (Campus Benjamin Franklin and Campus Virchow Klinikum, Charité—Universitätsmedizin Berlin; University of Bonn; Goethe-University Frankfurt; University of Cologne and University Hospital Heidelberg) between September 2009 and April 2018.^4^ The protocol was approved by the local ethics committees. Either informed consent or surrogate informed consent was obtained. Research was conducted in accordance with the Declaration of Helsinki. Results were reported following the STROBE guidelines (https://www.strobe-statement.org). DISCHARGE-1 was preregistered (http://www.isrctn.com/ISRCTN05667702). If the patient was eligible, a subdural electrode strip (Wyler, Ad-Tech Medical, Racine, WI, USA) for SD monitoring was placed over vital cortex.

In DISCHARGE-1, 180 of 205 (87.8%) patients could be analysed. For the present substudy, 44 additional patients were excluded because (i) the preoperative CT was missing (*n* = 18); (ii) the CT slice thickness was <3 mm or >6 mm (*n* = 3); (iii) the patient died early before the occurrence of delayed infarction could be assessed (*n* = 10); (iv) the patient experienced a periprocedural postoperative haemorrhage with a volume >10 ml (*n* = 8); (v) the first postoperative neuroimage showed malignant early brain injury (*n* = 2) or (vi) an interventional complication occurred such as infarction due to clip stenosis (*n* = 3). Placement of the electrode strip was performed either directly after surgical treatment of the aneurysm via craniotomy (*n* = 120) or, in coiled patients, after burr hole trepanation simultaneously with the placement of a ventricular drain or oxygen sensor (*n* = 16). All evaluators were blinded to other measures.

**Figure 1:**
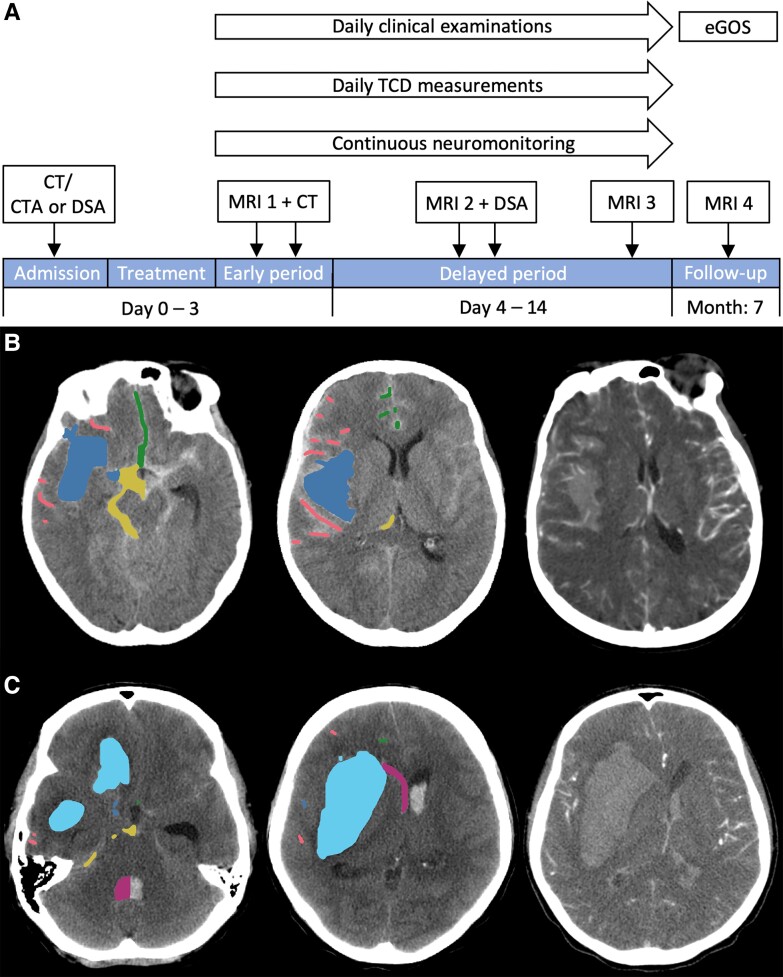
**Diagnostic flow of the DISCHARGE-1 study and quantification method for ipsilateral haemorrhage on the initial CT scan according to six predefined compartments.** (**A**) CT and CTA were performed on admission. If necessary, CTA was complemented by DSA. The first MRI (MRI 1) was acquired 24–48 h after surgical or endovascular treatment of the aneurysm. In addition, a postoperative CT was performed to locate the subdural electrodes. In the delayed period, MRI 2 was performed around Day 7 and MRI 3 around Day 14. Note that delayed infarct volumes were quantified for both sessions separately and then added up for further analysis. A second DSA was performed around Day 7 to assess angiographic vasospasm. After treatment of the aneurysm, the patient was transferred to the neurocritical care unit, where continuous neuromonitoring, daily TCD and clinical examinations started and continued until Day 14. After 7 months, a follow-up MRI 4 was performed. Furthermore, functional outcome was documented using the extended Glasgow Outcome Scale (eGOS). (**B**) Representative CT and CTA images of a patient with aSAH from a right MCA aneurysm. Note that haemorrhage was only quantified in the hemisphere ipsilateral to the subdural electrodes. CTA (right image) demonstrated contrast-enhancing vessels inside a large right-sided haematoma. Therefore, the volume of this haematoma was quantified according to the category of subarachnoid blood in the Sylvian fissure (blood_Sylvian_) (blue label in left and middle image). Blood_convex_ comprised blood in the sulci at the cerebral convexity including the rami of the Sylvian fissure (red label). Blood_inter_ was composed of blood in the anterior and posterior interhemispheric fissure as well as adjacent sulci (green label). Interhemispheric blood that crossed the midline was classified as contralateral and was not considered. Blood_basal_ included blood in the following cisterns: prepontine, interpeduncular, suprasellar, ipsilateral ambient, quadrigeminal and the interpositum cistern (yellow label). Subdural blood, seen as narrow hyperdense fringe overlying the left frontal cortex, was not quantified. (**C**) Representative CT and CTA images of another patient with aSAH from a right MCA aneurysm. CTA (right image) demonstrated contrast-enhancing vessels of the M2 segment of the MCA outside a large right-sided haematoma. Therefore, this haematoma volume was quantified within the ICH category (cyan label in left and middle image). Blood in the ventricular system is shown in purple. Most notably, a large clot was found in the fourth ventricle. This was only quantified until the midline (purple label). Blood_basal_ (yellow label), blood_inter_ (green label) and blood_convex_ (red label) were small in this patient.

The study design of DISCHARGE-1 has been previously described in great detail.^4^Figure 1A shows the study flow. In brief, neuroimaging included the pre-interventional CT to establish the diagnosis of aSAH and the post-interventional CT to locate the subdural ECoG electrodes. For the present substudy, early ischaemic cerebral infarcts were assessed using either a post-interventional MRI (*n* = 118) or CT (*n* = 18) performed no later than Day 5. The median day of this neuroimage, referred to as Image_early_, was Day 2 [interquartile range (IQR): 1–3]. Delayed ischaemic infarcts were assessed using a follow-up Image_late_ (MRI: *n* = 120, CT: *n* = 16) on Day 14 (IQR: 13–15) in comparison to Image_early_. Recording, analysis and interpretation of SDs followed the published recommendations of the Co-Operative Studies on Brain Injury Depolarisations (COSBID) group.^72^ Importantly, in every patient, the first 24-h period after the initial haemorrhage was always denoted as ‘Day 0’, the second 24-h period as ‘Day 1’ and so on. Using LabChart-8 software (ADInstruments, Bella Vista, New South Wales, Australia), M.K.L.W. and C.L.L. determined the following for each recording day of each patient: (i) total (cumulative) SD-induced depression duration (TDDD); (ii) number of SDs; (iii) number of SDs in electrically inactive tissue (isoelectric SDs)^72,73^ and (iv) number of clustered SDs, i.e. SDs that occurred less than 1 h apart from the previous SD. For the present substudy, we used peak values of a recording day for each SD-variable resulting in (i) PTDDD_delayed_; (ii) peak number of SDs of any type (peak_SD-delayed_); (iii) peak number of isoelectric SDs (peak_isoSD-delayed_) and (iv) peak number of clustered SDs (peak_clusSD-delayed_) for the delayed period between Image_early_ and Image_late_ after the end of neuromonitoring. TCD to determine mean blood flow velocities (mbfv) of the ACA (788 measurements), MCA (1060 measurements) and PCA (606 measurements) ipsilateral to the subdural electrodes was performed daily (*n* = 128). On this basis, peak values were determined for each of the three arteries and each patient. V.K. determined vascular narrowing using a qualitative grading score (no vascular narrowing = 1, vascular narrowing by 11–33% = 2, vascular narrowing by 34–66% = 3, vascular narrowing >67% = 4) for all DSAs performed between Days 5 and 17 [median Day 7 (IQR: 7–8), *n* = 106].^4^ The assessment included the first and second segments of MCA, ACA and PCA ipsilateral to the subdural electrodes (see also DSA grading score in the Supplementary material and Supplementary Fig. 1).

### Delayed cerebral infarcts

We adopted parenchymal lesion volumes derived from manual segmentation in DISCHARGE-1.^4^ For the present substudy, we only used delayed ischaemic infarct volumes in the cerebral hemisphere ipsilateral to the subdural electrodes. Following Weidauer *et al*.,^74^ the volumes of ipsilateral delayed infarction were further segmented into five categories: cortical ACA infarction, cortical MCA infarction, cortical PCA infarction, cortical watershed infarction and deep infarction. The latter included perforator infarcts, singular white matter infarcts without cortical involvement and anterior choroidal artery infarcts. Ischaemic tissue adjacent to the aneurysm was excluded. The location of delayed infarcts was determined according to arterial territory maps specified by Tatu *et al*.^75^

### Haemorrhage volumes

V.H. used the pre-interventional CT performed on median Day 0 (IQR: 0–0, range: 0–3) for volumetric haemorrhage quantification of the hemisphere ipsilateral to the subdural electrodes. Epidural, subdural, contralateral and infratentorial haemorrhages were not considered. Manual segmentation was carried out on non-contrast-enhanced images with a slice thickness between 3 and 6 mm using the paintbrush mode of ITK-Snap, Version 3.8.0 (www.itksnap.org). Ipsilateral haemorrhage was segmented into six predefined regions: subarachnoid blood accumulations on (i) the cerebral convexity (blood_convex_); (ii) in the interhemispheric fissure (blood_inter_); (iii) Sylvian fissure (blood_Sylvian_), or (iv) basal cisterns (blood_basal_); (v) ICH, or (vi) intraventricular haemorrhage (IVH) (see Fig. 1B and C). According to van der Zande *et al*.,^76^ we differentiated blood_Sylvian_ from ICH using CT angiography. Contrast-enhancing arteries within a haematoma indicated blood_Sylvian_, whereas a haematoma without visible contrast-enhancing vessels indicated ICH.

### Statistical analysis

The statistical analysis was performed by P.M., the trial statistician of DISCHARGE-1. Unless otherwise stated, data are given as median (IQR). Relationships between the variable groups related to blood volume, SD, DSA, TCD-determined peak mbfvs and delayed infarct volumes were analysed bivariately using Spearman correlations. Correlations with uncorrected *P*-values <0.05 were discussed. However, for each group of comparisons it was noted which correlations remained significant after Bonferroni correction. In the next step, principal component analyses were performed for each of the variable groups, and only the first principal component was used in further analyses. In principal component analyses, log transformations were applied for blood volume variables, SD variables, DSA variables and delayed infarct volumes, but not for TCD-determined peak mbfvs. The obtained components were included in path models with *a priori* defined structure, treating blood volume variables as extrinsic variables, SD variables, DSA variables and TCD-determined peak mbfvs as potential mediator variables but also possibly extrinsic variables, and infarct volumes due to DCI as outcome. Analyses were performed using SPSS for Windows release 26. The path models were calculated using Amos release 26.

### Data availability

Electronic recording, processing and storage of the data were approved by the data protection officer of the Charité—Universitätsmedizin Berlin (data protection votes from 28 May 2008 to 5 May 2014). The datasets analysed during the current study are not publicly available because the patient’s informed consent only permits the data analysis and publication by the investigators.

## Results

The DISCHARGE-1 cohort has been described previously.^4^ The present substudy included 90 (66.2%) females and 46 (33.8%) males. Median age was 56 (IQR: 47–63) years. All given data refer to the hemisphere ipsilateral to the subdural electrodes. Table 1 summarises the radiographic characteristics including haemorrhage and infarct volumes. The image_early_ revealed early infarcts in 80 (58.8%) patients with a total volume of 1156.3 ml. The image_late_ showed delayed infarcts in 69 (50.7%) patients with a total volume of 2599.7 ml. In addition, early and delayed infarcts are listed in Table 1 according to the five categories explained above. Because delayed cortical watershed infarcts and deep infarcts accounted for only a very small proportion of infarcts, they were not considered in further analyses. Table 1 also includes correlations of the composite delayed infarct volume in the ipsilateral ACA, MCA and PCA territories with various summary measures.

**Table 1:** Radiographic characteristics of the ipsilateral hemisphere

	Number of patients/total number of patients (%)	Mean volume ± standard deviation (ml)^a^	Minimum–maximum in single patients (ml)	Cumulative volume over all 136 patients (ml) (% of total cumulative volume)
blood_convex_	118/136 (86.8%)	2.7 ± 3.0	0–16.3	371.8 (8.9%)
blood_inter_	131/136 (96.3%)	3.5 ± 4.3	0–28.8	471.0 (11.3%)
blood_Sylvian_	133/136 (97.8%)	8.1 ± 13.1	0–78.2	1099.7 (26.4%)
blood_basal_	132/136 (97.1%)	4.0 ± 3.5	0–22.4	545.4 (13.1%)
ICH	39/136 (28.7%)	8.1 ± 20.5	0–101.4	1103.6 (26.4%)
IVH	109/136 (80.1%)	4.3 ± 10.6	0–70.1	581.3 (13.9%)
ECI_ACA_	37/136 (27.2%)	2.7 ± 11.4	0–105.0	370.5 (32.0%)
ECI_MCA_	33/136 (24.3%)	3.5 ± 17.1	0–186.0	472.6 (40.9%)
ECI_PCA_	8/136 (5.9%)	0.6 ± 4.3	0–46.7	87.9 (7.6%)
ECI_watershed_	12/136 (8.8%)	0.5 ± 3.1	0–34.6	63.1 (5.5%)
ECI_deep_	45/136 (33.1%)	1.2 ± 3.2	0–23.0	162.2 (14.0%)
DCI_ACA_	18/136 (13.2%)	2.9 ± 11.5	0–76.6	391.2 (15.0%)
DCI_MCA_	49/136 (36.0%)	13.5 ± 33.9	0–188.0	1839.4 (70.8%)
DCI_PCA_	9/136 (6.6%)	1.1 ± 6.0	0–50.0	145.3 (5.6%)
DCI_watershed_	7/136 (5.1%)	0.3 ± 1.8	0–16.2	43.2 (1.7%)
DCI_deep_	30/136 (22.1%)	1.3 ± 5.4	0–50.0	180.6 (6.9%)

	No vasospasm^b^	Mild^c^	Moderate^d^	Severe^e^
DSA_A1_	38/103	38/103	16/103	11/103
DSA_A2_	47/104	33/104	18/104	6/104
DSA_M1_	54/105	29/105	17/105	5/105
DSA_M2_	47/105	43/105	12/105	3/105
DSA_P1_	70/91	17/91	3/91	1/91
DSA_P2_	77/90	11/90	2/90	0/90

	Variable	Spearman coefficient	*P*-value	Number of patients
Sum of DCI_ACA + MCA + PCA_	Sum of blood_convex + inter + Sylvian + basal_	**0.21**	**0.015**	136
	Sum of blood_convex + inter + Sylvian + basal_ + IVH	**0.17**	**0.046**	136
	Average mbfv_ACA/MCA/PCA_	**0.25**	**0.004**	128
	DSA_A1–P2_ score	**0.26**	**0.008**	106
	PTDDD_delayed_	**0.55**	**<0.001**	136
	peak_SD-delayed_	**0.46**	**<0.001**	136
	peak_isoSD-delayed_	**0.47**	**<0.001**	136
	peak_clusSD-delayed_	**0.43**	**<0.001**	136

Statistically significant values are marked in bold.

All given data only refer to the hemisphere ipsilateral to the subdural electrodes.

Average mbfv_ACA/MCA/PCA_ = average of the peak mean blood flow velocities of anterior cerebral artery (ACA), middle cerebral artery (MCA) and posterior cerebral artery (PCA); blood_basal_ = subarachnoid blood volume in the basal cisterns; blood_convex_ = subarachnoid blood volume on the cerebral convexity; blood_inter_ = subarachnoid blood volume in the interhemispheric fissure; blood_Sylvian_ = subarachnoid blood volume in the Sylvian fissure; DCI_ACA_ = delayed infarct volume in the territory of the ACA; DCI_deep_ = delayed infarct volume below the cortex including perforator infarcts, singular white matter infarcts without cortical involvement and anterior choroidal artery infarcts; DCI_MCA_ = delayed infarct volume in the territory of the MCA; DCI_PCA_ = delayed infarct volume in the territory of the PCA; DCI_watershed_ = delayed infarct volume in the territory of the cortical watershed zones; DSA = digital subtraction angiography (A1, A2, M1, M2, P1, P2 = first and second segments of ACA, MCA and PCA ipsilateral to the subdural electrodes); DSA_A1–P2_ score = total score achieved by the summation of values for A1, A2, M1, M2, P1 and P2 divided by the number of vessel segments assessed; ECI_ACA_ = early infarct volume in the territory of the anterior cerebral artery; ECI_deep_ = early infarct volume below the cortex including perforator infarcts, singular white matter infarcts without cortical involvement and anterior choroidal artery infarcts; ECI_MCA_ = early infarct volume in the territory of the middle cerebral artery; ECI_PCA_ = early infarct volume in the territory of the posterior cerebral artery; ECI_watershed_ = early infarct volume in the territory of the cortical watershed zones; ICH = intracerebral haemorrhage;

IVH = intraventricular haemorrhage; peak_clusSD-delayed_ = peak number of clustered spreading depolarizations (SD) of a recording day during the delayed period between the early post-intervention neuroimage and the late neuroimage after completion of neuromonitoring (clustered SD = SD that occurred less than 1 h apart from the previous SD); peak_isoSD-delayed_ = peak number of isoelectric SDs of a recording day during the delayed period (isoelectric SD = SD in electrically inactive tissue); peak_SD-delayed_ = peak number of SDs of any type of a recording day during the delayed period; PTDDD_delayed_ = peak value of a recording day for the total (cumulative) SD-induced depression durations during the delayed period; sum of blood_convex + inter + Sylvian + basal_ = total subarachnoid blood volume (blood_convex_ + blood_inter_ + blood_Sylvian_ + blood_basal_); sum of blood_convex + inter + Sylvian + basal_ + IVH = blood_convex_ + blood_inter_ + blood_Sylvian_ + blood_basal_ + IVH.

In the bottom part of the table, we added the delayed infarct volumes DCI_ACA_ + DCI_MCA_ + DCI_PCA_ and correlated this composite infarct volume with several summary measures, namely, the total subarachnoid blood volume (blood_convex_ + blood_inter_ + blood_Sylvian_ + blood_basal_) with and without IVH volume, the average of mbfv_ACA_ + mbfv_MCA_ + and mbfv_PCA_, the average score based on DSA_A1–P2_, and the four SD variables to provide an overview. Large subarachnoid haematoma with space-occupying effect and perifocal oedema are not listed separately in this table but are included in blood_inter_ and blood_Sylvian_. In total, we encountered 22 (16.2%) such cases with large subarachnoid haematomas with a median blood volume of 29 (IQR: 16–46) ml, 19 cases in the Sylvian fissure and 3 cases in the interhemispheric fissure.

a Zero values included.

b Vessel narrowing <11%.

c Vessel narrowing 11–33%.

d Vessel narrowing 34–66%.

e Vessel narrowing >66%.

### Illustrative case

**Figure 2:**
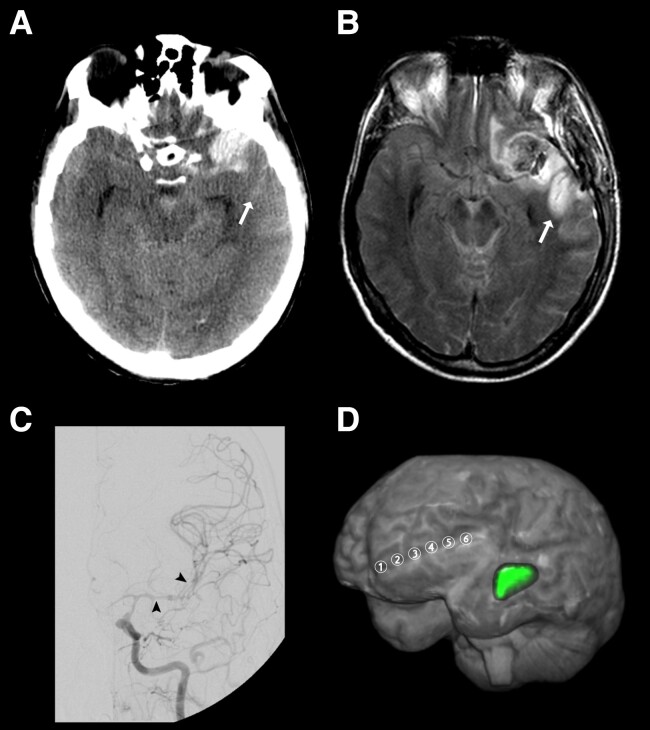
**Example case illustrating delayed cerebral infarction adjacent to blood on the cerebral convexity that was associated with a cluster of SDs and angiographic vasospasm.** (**A**) Representative CT image of the initial scan at the skull base. The initial scan was performed on Day 1 after the initial haemorrhage. Linear hyperdense abnormalities were observed in the sulci of the left cerebral convexity consistent with SAH that extensively covered the cortical surface (10.6 ml). Note that the anterior portion of the left superior temporal sulcus was also filled with blood (arrow). Furthermore, the left MCA aneurysm was surrounded by a hyperdense mass at the left temporal pole consistent with perianeurysmal haematoma that extended into cerebral parenchyma (11.8 ml). Only small to moderate amounts of blood were found in the Sylvian fissure (5.5 ml), the basal cisterns (4.5 ml), the interhemispheric fissure (1.2 ml) and in the ventricles (1.1 ml). (**B**) Representative FLAIR image of Image_late_ on Day 9 at the skull base. A new hyperintense signal was observed in the anterior portion of the superior temporal sulcus (arrow). The corresponding area showed hyperintensity on diffusion-weighted images and hypointensity on the apparent diffusion coefficient (ADC) map. These findings suggested a new delayed infarct in the temporal MCA territory adjacent to the sulcal blood clot seen on the initial CT scan (arrow). (**C**) The left angiogram on Day 9 revealed severe vasospasm in the intracranial segment of the internal carotid artery, the A1, M1 and M2 segments (see arrowheads for left MCA vasospasm). (**D**) The 3D visualization depicts the spatial relationship between the delayed MCA infarct (green label) and the electrode strip (electrodes 1–6). The strip was located on the left frontolateral cortex, whereas the delayed infarct evolved in the left temporal cortex. The shortest distance was measured between the infarct boundary and electrode 6. It amounted to 28 mm.

**Figure 3:**
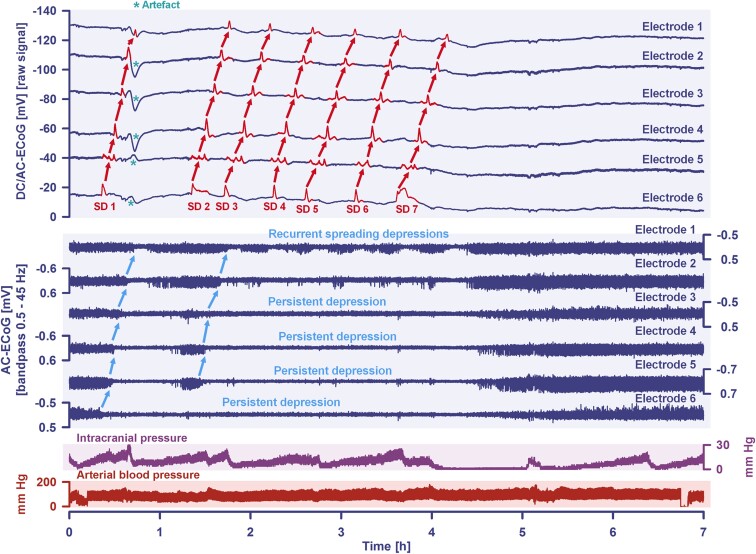
**A cluster of seven SDs is shown that occurred in the same patient as in Fig. 2 during a period of 4 h on Day 7 after the initial haemorrhage.** Traces 1–6 from top to bottom give the DC/AC-ECoG recordings (band-pass: 0–45 Hz). SDs are observed as a negative DC shift (marked in red in the traces). The SDs propagated across the cortex from electrode 6 to electrode 1. The direction of the propagation (shown by the red arrows) suggests that the SDs originated in an area closer to electrode 6 than to electrode 1. An artefact that likely relates to a systemic change in partial pressure of oxygen is marked with an asterisk after the first SD in traces 2–6. The following six traces (7–12) show the depressive effect of the SDs on the spontaneous brain activity as assessed in the high frequency band (AC-ECoG, band-pass: 0.5–45 Hz). Note that the activity depression propagates together with the SDs in the tissue (blue arrows). The spontaneous activity recovers after each SD only in electrode 1 (trace 7) and partially in electrode 2 (trace 8). In contrast, a persistent depression of activity is observed after the second SD in electrodes 3–5 (traces 9–11) and after the first SD in electrode 6 (trace 12). Thus, SDs 2–7 propagate in electrically silent tissue and are classified accordingly as isoelectric SDs.^72^ Of note, the longest SD-induced activity depression is found in trace 12 closer to the origin of SDs. Trace 13 shows the intracranial pressure measured via extraventricular drainage catheter. Trace 14 shows the systemic arterial pressure (measured via radial artery catheter).

A 48-year-old man was admitted to the emergency room after a seizure and continued loss of consciousness. The initial CT scan demonstrated Grade 4 aSAH (modified Fisher scale) (Fig. 2A) due to rupture of a DSA-proven aneurysm at the left MCA bifurcation. On Day 1, the aneurysm was secured by surgical clip ligation and a subdural electrode strip was placed. On Day 2, MRI showed no early cerebral infarction. Due to the reduced level of consciousness, neurological assessment was limited during neurocritical care. On Day 7, an intense SD cluster suddenly began (TDDD: 324.0 min) (Fig. 3). Figure 2B gives a fluid-attenuated inversion recovery (FLAIR) image on Day 9 that revealed a new hyperintense lesion in the left temporal cortex consistent with delayed cerebral infarction in the left MCA territory. DSA on the same day showed severe angiographic vasospasm (Fig. 2C). Daily TCD examinations of the ipsilateral MCA demonstrated increased mbfvs on 2 days (>120 cm/s), but the peak mbfv of 144 cm/s on Day 8 did not reach the critical threshold of 200 cm/s.^77^Figure 2D visualises the spatial relation between delayed MCA infarct and location of the subdural electrodes.

### Correlation analysis

**Figure 4:**
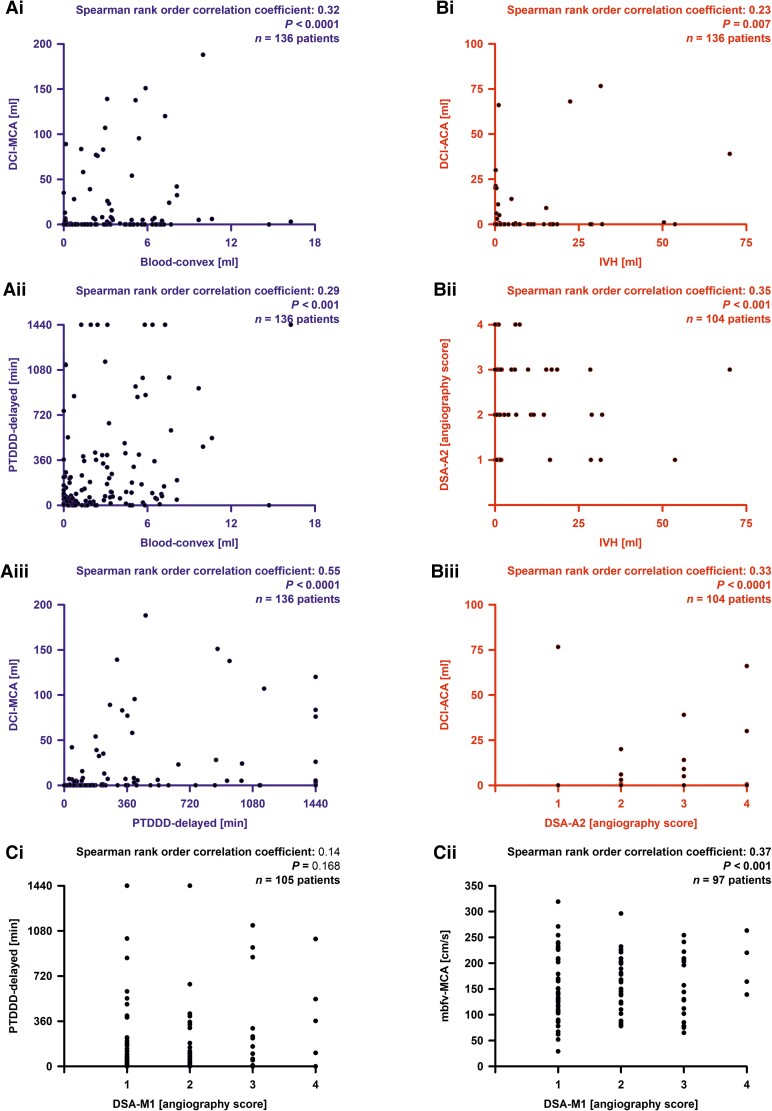
**Correlation analyses.** In the principal component and path analyses, we found two paths, one from extravascular blood volume component (blood_component_) to delayed cerebral ischaemia component (DCI_component_) with spreading depolarization component (SD_component_) as mediator variable, and one from IVH to DCI_component_ with DSA component as mediator variable. **(A**) Three strongest correlations between individual variables from the three variable groups involved in the path from blood_component_ to SD_component_ to DCI_component_. (**B**) Three strongest correlations between individual variables from the three variable groups involved in the path from IVH to DSA_component_ to DCI_component_. (**Ci**) The electrode strip was typically located on the cortex of the territory of the MCA. However, there was no correlation between angiographic vasospasm in the M1 segment (or M2 segment, see Supplementary Table 1) of the MCA (DSA_M1_) with the SD variables [shown here is the PTDDD_delayed_ (peak value of a recording day for the total (cumulative) SD-induced depression durations during the delayed period)]. (**Cii**) In contrast, the TCD-determined peak mean blood flow velocity of the MCA (mbfv_MCA_) correlated with DSA_M1_. blood_convex_ = subarachnoid blood volume on the cerebral convexity; DCI_ACA_ = delayed infarct volume in the territory of the anterior cerebral artery (ACA); DCI_MCA_ = delayed infarct volume in the territory of the MCA; DSA_A2_ = DSA score of the A2 segment of the ACA.

Our basic hypothesis was that certain blood volumes [(i) blood_convex_; (ii) blood_inter_; (iii) blood_Sylvian_; (iv) blood_basal_; (v) ICH and (vi) IVH] are associated with delayed infarct volumes. To this aim, we calculated Spearman correlations with delayed infarct volumes in the territories of ACA (DCI_ACA_), MCA (DCI_MCA_) and PCA (DCI_PCA_) (Table 2). We found correlations between blood_convex_ and DCI_MCA_ (*r* = 0.32, *P* < 0.001), blood_inter_ and DCI_ACA_ (*r* = 0.19, *P* = 0.030), blood_Sylvian_ and DCI_MCA_ (*r* = 0.25, *P* = 0.003), blood_basal_ and DCI_MCA_ (*r* = 0.23, *P* = 0.008), and IVH and DCI_ACA_ (*r* = 0.23, *P* = 0.007) (Fig. 4Bi). Applying a Bonferroni correction with factor 18 (six blood volumes, three delayed infarct variables), the correlation between blood_convex_ and DCI_MCA_ remained significant. Thus, the basic hypothesis was proven for blood_convex_ and DCI_MCA_ (Fig. 4Ai).

**Table 2:** Spearman correlations between region-specific blood volumes and delayed cortical infarct volumes

Statistical analysis	Variable	Spearman coefficient	*P*-value	Number of patients
Association analysis with DCI_ACA_	blood_convex_	−0.03	0.691	136
	blood_inter_	**0**.**19**	**0**.**030**	136
	blood_Sylvian_	0.01	0.899	136
	blood_basal_	0.07	0.409	136
	ICH	0.07	0.418	136
	IVH	**0**.**23**	**0**.**007**	136
Association analysis with DCI_MCA_	blood_convex_	**0**.**32**	**<0.001**	136
	blood_inter_	0.09	0.297	136
	blood_Sylvian_	**0**.**25**	**0**.**003**	136
	blood_basal_	**0**.**23**	**0**.**008**	136
	ICH	0.07	0.420	136
	IVH	0.12	0.181	136
Association analysis with DCI_PCA_	blood_convex_	0.10	0.269	136
	blood_inter_	−0.05	0.572	136
	blood_Sylvian_	0.03	0.704	136
	blood_basal_	0.01	0.890	136
	ICH	0.10	0.268	136
	IVH	0.15	0.085	136

Statistically significant values are marked in bold.

All given data only refer to the hemisphere ipsilateral to the subdural electrodes. blood_basal_ = subarachnoid blood volume in the basal cisterns; blood_convex_ = subarachnoid blood volume on the cerebral convexity; blood_inter_ = subarachnoid blood volume in the interhemispheric fissure; blood_Sylvian_ = subarachnoid blood volume in the Sylvian fissure; DCI_ACA_ = delayed infarct volume in the territory of the anterior cerebral artery; DCI_MCA_ = delayed infarct volume in the territory of the middle cerebral artery; DCI_PCA_ = delayed infarct volume in the territory of the posterior cerebral artery; ICH = intracerebral haemorrhage; IVH = intraventricular haemorrhage.

Then, we investigated the role of potential mediator variables (SD variables, TCD-determined peak mbfvs and DSA variables), which should be associated with blood volume variables (Table 3) and delayed infarct volume variables (Table 4). First, we investigated the correlations between blood volumes and SD variables. Because the four SD variables were highly correlated with each other, there was a clear pattern: Blood_convex_ (correlations between 0.24 and 0.29) (Fig. 4Aii) and blood_Sylvian_ (correlations between 0.21 and 0.30) were correlated with each SD variable, whereas blood_inter_, blood_basal_, ICH and IVH were not. Applying a Bonferroni correction with factor 24 (six blood volumes, four SD variables), four of these eight correlations remained significant. Furthermore, SD variables were correlated with each of the delayed infarct volume variables. Correlations of SD variables were larger with DCI_MCA_ (0.46–0.55) (Fig. 4Aiii) and smaller with DCI_ACA_ (0.18–0.23) and DCI_PCA_ (0.19–0.26). After Bonferroni correction with factor 12 (four SD variables, three delayed infarct volume variables), each of the four correlations between SD variables and DCI_MCA_ remained significant. Thus, using the assumption that SD variables are in the pathway between blood volume variables and delayed infarct volume variables, the role of a mediator was supported by the correlation analyses. A more precise analysis is presented in the ‘Path analysis’ section.

**Table 3:** Spearman correlations between region-specific blood volumes and potential mediators of delayed infarction

Statistical analysis	Variable	Spearman coefficient	*P*-value	Number of patients
Association analysis with blood_convex_	PTDDD_delayed_	**0**.**29**	**0**.**001**	136
	peak_SD-delayed_	**0**.**25**	**0**.**003**	136
	peak_isoSD-delayed_	**0**.**25**	**0**.**003**	136
	peak_clusSD-delayed_	**0**.**24**	**0**.**005**	136
	mbfv_ACA_	0.11	0.265	112
	mbfv_MCA_	**0**.**21**	**0**.**020**	128
	mbfv_PCA_	0.08	0.442	98
	DSA_A1_	−0.01	0.915	103
	DSA_A2_	−0.14	0.165	104
	DSA_M1_	0.10	0.302	105
	DSA_M2_	**0**.**24**	**0**.**014**	105
	DSA_P1_	0.05	0.633	91
	DSA_P2_	0.02	0.842	90
Association analysis with blood_inter_	PTDDD_delayed_	0.08	0.342	136
	peak_SD-delayed_	0.04	0.647	136
	peak_isoSD-delayed_	0.05	0.542	136
	peak_clusSD-delayed_	0.03	0.716	136
	mbfv_ACA_	−0.08	0.420	112
	mbfv_MCA_	0.00	0.993	128
	mbfv_PCA_	0.13	0.215	98
	DSA_A1_	**0**.**21**	**0**.**032**	103
	DSA_A2_	**0**.**31**	**0**.**001**	104
	DSA_M1_	0.09	0.345	105
	DSA_M2_	−0.07	0.454	105
	DSA_P1_	−0.01	0.957	91
	DSA_P2_	0.05	0.677	90
Association analysis with blood_Sylvian_	PTDDD_delayed_	**0**.**28**	**0**.**001**	136
	peak_SD-delayed_	**0**.**25**	**0**.**004**	136
	peak_isoSD-delayed_	**0**.**30**	**<0.001**	136
	peak_clusSD-delayed_	**0**.**21**	**0**.**016**	136
	mbfv_ACA_	−0.02	0.843	112
	mbfv_MCA_	0.11	0.215	128
	mbfv_PCA_	0.01	0.930	98
	DSA_A1_	−0.06	0.560	103
	DSA_A2_	−0.11	0.281	104
	DSA_M1_	−0.04	0.654	105
	DSA_M2_	0.15	0.126	105
	DSA_P1_	0.05	0.663	91
	DSA_P2_	0.07	0.514	90
Association analysis with blood_basal_	PTDDD_delayed_	0.14	0.100	136
	peak_SD-delayed_	0.09	0.292	136
	peak_isoSD-delayed_	0.15	0.088	136
	peak_clusSD-delayed_	0.08	0.331	136
	mbfv_ACA_	−0.07	0.491	112
	mbfv_MCA_	0.06	0.525	128
	mbfv_PCA_	0.16	0.113	98
	DSA_A1_	0.16	0.097	103
	DSA_A2_	**0**.**20**	**0**.**043**	104
	DSA_M1_	0.03	0.761	105
	DSA_M2_	−0.02	0.876	105
	DSA_P1_	0.08	0.453	91
	DSA_P2_	0.16	0.125	90
Association analysis with ICH	PTDDD_delayed_	0.10	0.274	136
	peak_SD-delayed_	0.07	0.413	136
	peak_isoSD-delayed_	0.04	0.622	136
	peak_clusSD-delayed_	0.06	0.500	136
	mbfv_ACA_	0.08	0.413	112
	mbfv_MCA_	−0.03	0.703	128
	mbfv_PCA_	0.03	0.806	98
	DSA_A1_	0.08	0.419	103
	DSA_A2_	0.06	0.548	104
	DSA_M1_	0.14	0.157	105
	DSA_M2_	0.04	0.697	105
	DSA_P1_	0.08	0.450	91
	DSA_P2_	0.04	0.691	90
Association analysis with IVH	PTDDD_delayed_	0.05	0.551	136
	peak_SD-delayed_	0.00	0.977	136
	peak_isoSD-delayed_	−0.03	0.734	136
	peak_clusSD-delayed_	0.03	0.718	136
	mbfv_ACA_	0.10	0.290	112
	mbfv_MCA_	0.08	0.399	128
	mbfv_PCA_	0.16	0.127	98
	DSA_A1_	0.19	0.058	103
	DSA_A2_	**0**.**35**	**<0.001**	104
	DSA_M1_	0.17	0.088	105
	DSA_M2_	**0**.**20**	**0**.**041**	105
	DSA_P1_	**0**.**31**	**0**.**003**	91
	DSA_P2_	**0**.**26**	**0**.**015**	90

Statistically significant values are marked in bold.

All given data only refer to the hemisphere ipsilateral to the subdural electrodes. blood_basal_ = subarachnoid blood volume in the basal cisterns; blood_convex_ = subarachnoid blood volume on the cerebral convexity; blood_inter_ = subarachnoid blood volume in the interhemispheric fissure; blood_Sylvian_ = subarachnoid blood volume in the Sylvian fissure; DSA = digital subtraction angiography [A1, A2, M1, M2, P1, P2 = first and second segments of anterior cerebral artery (ACA), middle cerebral artery (MCA) and posterior cerebral artery (PCA) ipsilateral to the subdural electrodes]; ICH = intracerebral haemorrhage; IVH = intraventricular haemorrhage; mbfv_ACA_ = transcranial Doppler-sonography (TCD)-determined peak mean blood flow velocity of ACA; mbfv_MCA_ = TCD-determined peak mean blood flow velocity of MCA; mbfv_PCA_ = TCD-determined peak mean blood flow velocity of PCA; peak_clusSD-delayed_ = peak number of clustered spreading depolarizations (SD) of a recording day during the delayed period between the early post-intervention neuroimage and the late neuroimage after completion of neuromonitoring (clustered SD = SD that occurred less than 1 h apart from the previous SD); peak_isoSD-delayed_ = peak number of isoelectric SDs of a recording day during the delayed period (isoelectric SD = SD in electrically inactive tissue); peak_SD-delayed_ = peak number of SDs of any type of a recording day during the delayed period; PTDDD_delayed_ = peak value of a recording day for the total (cumulative) SD-induced depression durations during the delayed period.

**Table 4:** Spearman correlations between potential mediators and delayed cortical infarct volumes

Statistical analysis	Variable	Spearman coefficient	*P*-value	Number of patients
Association analysis with DCI_ACA_	PTDDD_delayed_	**0**.**19**	**0**.**024**	136
	peak_SD-delayed_	**0**.**18**	**0**.**042**	136
	peak_isoSD-delayed_	**0**.**23**	**0**.**006**	136
	peak_clusSD-delayed_	**0**.**18**	**0**.**033**	136
	mbfv_ACA_	0.15	0.126	112
	mbfv_MCA_	0.16	0.076	128
	mbfv_PCA_	0.01	0.936	98
	DSA_A1_	**0**.**23**	**0**.**017**	103
	DSA_A2_	**0**.**33**	**0**.**001**	104
	DSA_M1_	**0**.**22**	**0**.**025**	105
	DSA_M2_	**0**.**21**	**0**.**031**	105
	DSA_P1_	**0**.**33**	**0**.**002**	91
	DSA_P2_	**0**.**31**	**0**.**003**	90
Association analysis with DCI_MCA_	PTDDD_delayed_	**0**.**55**	**<0.001**	136
	peak_SD-delayed_	**0**.**47**	**<0.001**	136
	peak_isoSD-delayed_	**0**.**50**	**<0.001**	136
	peak_clusSD-delayed_	**0**.**46**	**<0.001**	136
	mbfv_ACA_	**0**.**24**	**0**.**010**	112
	mbfv_MCA_	**0**.**20**	**0**.**021**	128
	mbfv_PCA_	**0**.**22**	**0**.**031**	98
	DSA_A1_	**0**.**21**	**0**.**030**	103
	DSA_A2_	0.10	0.318	104
	DSA_M1_	**0**.**25**	**0**.**012**	105
	DSA_M2_	0.16	0.099	105
	DSA_P1_	0.08	0.456	91
	DSA_P2_	0.12	0.257	90
Association analysis with DCI_PCA_	PTDDD_delayed_	**0**.**22**	**0**.**012**	136
	peak_SD-delayed_	**0**.**26**	**0**.**003**	136
	peak_isoSD-delayed_	**0**.**24**	**0**.**006**	136
	peak_clusSD-delayed_	**0**.**19**	**0**.**031**	136
	mbfv_ACA_	**0**.**32**	**<0.001**	112
	mbfv_MCA_	0.12	0.172	128
	mbfv_PCA_	0.19	0.056	98
	DSA_A1_	0.11	0.265	103
	DSA_A2_	0.15	0.122	104
	DSA_M1_	0.06	0.563	105
	DSA_M2_	−0.02	0.874	105
	DSA_P1_	−0.02	0.868	91
	DSA_P2_	0.04	0.700	90

Statistically significant values are marked in bold.

All given data only refer to the hemisphere ipsilateral to the subdural electrodes. DCI_ACA_ = delayed infarct volume in the territory of the anterior cerebral artery; DCI_MCA_ = delayed infarct volume in the territory of the middle cerebral artery; DCI_PCA_ = delayed infarct volume in the territory of the posterior cerebral artery; DSA = digital subtraction angiography (A1, A2, M1, M2, P1, P2 = first and second segments of ACA, MCA and PCA ipsilateral to the subdural electrodes); ICH = intracerebral haemorrhage; IVH = intraventricular haemorrhage; mbfv_ACA_ = transcranial Doppler-sonography (TCD)-determined peak mean blood flow velocity of ACA; mbfv_MCA_ = TCD-determined peak mean blood flow velocity of MCA; mbfv_PCA_ = TCD-determined peak mean blood flow velocity of PCA; peak_clusSD-delayed_ = peak number of clustered spreading depolarizations (SD) of a recording day during the delayed period between the early post-intervention neuroimage and the late neuroimage after completion of neuromonitoring (clustered SD = SD that occurred less than 1 h apart from the previous SD); peak_isoSD-delayed_ = peak number of isoelectric SDs of a recording day during the delayed period (isoelectric SD = SD in electrically inactive tissue); peak_SD-delayed_ = peak number of SDs of any type of a recording day during the delayed period; PTDDD_delayed_ = peak value of a recording day for the total (cumulative) SD-induced depression durations during the delayed period.

Applying the same procedure to peak mbfvs, we only found one correlation between blood_convex_ and mbfv_MCA_ (*r* = 0.21, *P* = 0.02), which was not significant after Bonferroni correction with factor 18 (six blood volume variables, three peak mbfvs for MCA, ACA and PCA). Of nine correlations between peak mbfvs and delayed infarct variables, four had uncorrected *P*-values smaller than 0.05 [mbfv_MCA_ with DCI_MCA_, (*r* = 0.20, *P* = 0.02), mbfv_ACA_ with DCI_MCA_ (*r* = 0.24, *P* = 0.01), mbfv_ACA_ with DCI_PCA_ (*r* = 0.32, *P* < 0.001) and mbfv_PCA_ with DCI_MCA_ (*r* = 0.22, *P* = 0.03)]. The correlation between mbfv_ACA_ and DCI_PCA_ remained significant after Bonferroni correction. We concluded that peak mbfvs were not a mediator variable although they were associated with delayed infarcts. Based on these results, we did not further examine this variable in the path analysis. Regarding DSA variables, we found eight correlations, one between blood_convex_ and DSA_M2_, two between blood_inter_ and DSA_A1_ and DSA_A2_, none between blood_Sylvian_ or ICH and any DSA variable, one between blood_basal_ and DSA_A2_, and four between IVH and DSA_M2_, DSA_A2_, DSA_P1_ and DSA_P2_. Two of these correlations, blood_inter_ with DSA_A2_ (*r* = 0.31, *P* = 0.001) and IVH with DSA_A2_ (*r* = 0.35, *P* = 0.001) (Fig. 4Bii) remained significant after Bonferroni correction with factor 36 (six blood volumes and six DSA variables). Furthermore, we found eight correlations between DSA variables and delayed infarct volume variables. Each of the DSA variables was correlated with DCI_ACA_, and additionally, DSA_M1_ and DSA_A1_ with DCI_MCA_. After Bonferroni correction with factor 18 (six DSA variables, three delayed infarct variables), the correlations of DSA_A2_ with DCI_ACA_ (*r* = 0.33, *P* = 0.001) (Fig. 4Biii), and of DSA_P1_ with DCI_ACA_ (*r* = 0.33, *P* = 0.002) remained significant. Therefore, we considered angiographic vasospasm as a potential mediator in the path analysis.

### Principal component and path analysis

**Figure 5:**
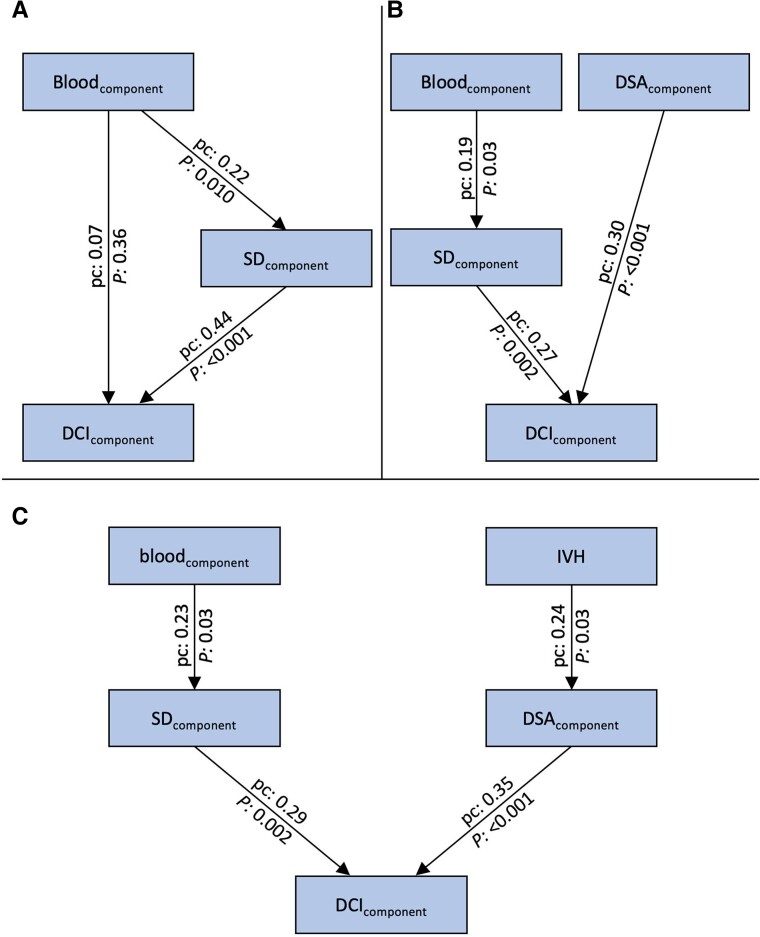
**Path models.** (A) The first principal component of the SD variables (SD_component_) mediates the effect of the blood volume variables (blood_component_) on delayed infarct volumes (DCI_component_). **(B)** Path model treating blood_component_ and the first principal component of the DSA variables (DSA_component_) as extrinsic variables and SD_component_ as mediator variable. **(C)** Path model including IVH into the analysis. There were two paths, one from blood_component_ to DCI_component_ with SD_component_ as mediator variable, and one from IVH to DCI_component_ with DSA_component_ as mediator variable. The numbers at the arrows represent the pc and the *P*-values (the corresponding z-values are found in the text). *P*-values of path models use the standard normal distribution for quotients of unstandardised pc and their standard errors and Chi-square tests for model fit with degrees of freedom equal to ‘number of parameters saturated model minus number of parameters actual model’.^78^

For each group of variables, the first principal component was used in the path analysis. We refrained from calculating a structural equation model, as single variables were far from normally distributed, although the principal components of blood volume (blood_component_), SD variables (SD_component_), peak mbfvs (mbfv_component_) and angiographic vasospasm (DSA_component_) were close to normal distribution. In the first path model, we only included SD variables, as there were many missing values for peak mbfvs and DSA variables and our primary interest was to investigate the potential role of SDs. Standardised path coefficients (pc) were 0.22 for the path from blood_component_ to SD_component_ (*P* = 0.010, z = 2.56); and 0.44 for the path from SD_component_ to the first principal component of delayed infarct volume (DCI_component_) (*P* < 0.001, z = 5.54); but only 0.07 for the direct path from blood_component_ to DCI_component_ (*P* = 0.36, z = 0.91) (Fig. 5A). Thus, the role of SDs as a mediator between blood volume and delayed infarct volume was confirmed.

For DSA variables, the path from DSA_component_ to DCI_component_ had a standardised pc of 0.37 (*P* < 0.001, z = 3.67). However, the pc was only 0.12 (*P* = 0.28, z = 1.08) for the path from blood_component_ to DSA_component_ which questions the role of angiographic vasospasm as a mediator variable between blood volume and delayed infarct volume, although there was a clear association between angiographic vasospasm and delayed infarct volume. For mbfv_component_, pc were all <0.20. Therefore, this component was not included in further analyses.

Based on these analyses, we constructed a path model with the extrinsic variables blood volume and angiographic vasospasm, one mediator variable (SD) and the outcome variable delayed infarct volume. In this model, pc did not change considerably compared to the separate analyses for SD and angiographic vasospasm: There was a path from blood_component_ to SD_component_ (pc = 0.19, *P* = 0.03, z = 2.17), and from SD_component_ to DCI_component_ (pc = 0.27, *P* = 0.002, z = 3.06), and a direct path from DSA_component_ to DCI_component_ (pc = 0.30, *P* < 0.001, z = 3.65). The model showed an excellent fit (Chi-Square = 1.8, degrees of freedom = 3, *P* = 0.61) (Fig. 5B).

In the principal component analysis, IVH was not represented in the first component. Thus, based on the correlation analyses, we constructed a second path model with the principal component of blood volume without IVH as first and IVH as second extrinsic variable. There were two paths, one from blood_component_ to DCI_component_ with SD_component_ as mediator variable (pc from blood_component_ to SD_component_ = 0.23, *P* = 0.03, z = 2.17; pc from SD_component_ to DCI_component_ = 0.29, *P* = 0.002, z = 3.06), and one from IVH to DCI_component_ with DSA_component_ as mediator variable (pc from IVH to DSA_component_ = 0.24, *P* = 0.03, z = 2.17; pc from DSA_component_ to DCI_component_ = 0.35, *P* < 0.001, z = 3.65). No additional paths in this model were significant. This model also showed an excellent fit (Chi-Square = 5.3, degrees of freedom = 6, *P* = 0.51) (Fig. 5C).

### Further associations

Supplementary Table 1 shows correlations between SD variables, DSA variables and peak mbfvs. SD and DSA variables did not correlate (Fig. 4Ci). Of 12 correlations between SD variables and peak mbfvs, 4 had uncorrected *P*-values smaller than 0.05. None of these remained significant after Bonferroni correction. Of 18 correlations between peak mbfvs and DSA variables, 4 had uncorrected *P*-values smaller than 0.05. After Bonferroni correction with factor 18 (three mbfvs, six DSA variables), the correlation between mbfv_MCA_ with DSA_M1_ remained significant (Fig. 4Cii).

### Discussion

It is assumed that the amount of subarachnoid blood on the initial CT scan predicts DCI.^1^Supplementary Table 2 lists the studies we found in which blood was quantified and all studies supported this.^79-84^ Our study basically reaches the same conclusion. However, we also quantified blood in the sulci of the cerebral convexity, and this component had the strongest statistical association with delayed infarcts in the MCA territory, which in turn accounted for 70.8% of the total cumulative infarct volume in the 136 patients. In fact, only the correlation between blood_convex_ and DCI_MCA_ remained significant with strict Bonferroni correction. However, with 18 tests, only 1 uncorrected significant result is expected by chance, and we observed such significances in 5 tests (Table 2). Four of these were related to the same fundamental hypothesis—local blood deposition on the cortex contributes to delayed infarct pathogenesis. Therefore, we estimate Bonferroni correction to be very conservative here and believe that the fundamental hypothesis above is also supported by the additional tests, which were significant without Bonferroni correction. For example, blood_inter_ without Bonferroni correction correlated significantly with delayed infarcts in the ACA territory adjacent to the interhemispheric fissure, and blood_Sylvian_ showed the second strongest correlation with delayed infarcts in the MCA territory surrounding the Sylvian fissure.

Human autopsy studies shaped the hypothesis that local blood deposition on the cortex is largely responsible for infarcts after aSAH,^55^ which is further supported by radiological findings^61,85-87^ and a primate study.^63^ This hypothesis implies that direct exposure to factors released from the clot is critically involved in cortical infarct development below the clot. Experimentally, an important effect of such factors is to induce SDs, which in turn initiate and maintain neuronal cytotoxic oedema associated with the risk of developing into infarction. Consistently, focal accumulation of subarachnoid blood was a sufficient insult to trigger SDs and early infarcts in a swine model.^47^ SD induction was also previously demonstrated in a rat model mimicking post-aSAH conditions.^21^ In this model, artificial cerebrospinal fluid (aCSF), with an increased K^+^ concentration ([K^+^]_aCSF_) and either a nitric oxide synthase (NOS) inhibitor or the nitric oxide (NO) scavenger haemoglobin, was applied topically on the brain.^21^ The same protocol also induced SDs in brain slices devoid of intact blood circulation.^39^ For the complex role of K^+^, the reader is referred to previous work.^5,88^ The prominent role of decreased NO availability agrees well with the increasingly recognised hypothesis, originally from Furchgott *et al*., that clot-derived factors cause NO deficiency after aSAH.^57,58,89,90^ NO deficiency leads directly to vasoconstriction and, by absence of its permissive effect for other vasodilators, indirectly as well.^5,58^ NO deficiency also lowers the SD threshold. This was found not only *in vivo*^24^ but also in brain slices^39^ devoid of intact blood circulation. Previous work suggested that loss of cyclic guanosine monophosphate (cGMP)-independent modulatory effects of NO on neuronal P/Q-type voltage-gated Ca^2+^ channels and *N*-methyl-D-aspartate receptor-controlled channels are responsible for this.^91^ However, even in absence of NO-lowering agents, increased microvascular tone can cause SDs due to an imbalance between energy supply and demand of neurons. This was demonstrated in an *in vivo* model with ascending epipially applied concentrations of the vasoconstrictor polypeptide endothelin-1, which failed in brain slices.^92^ There are both arguments in favour of and against vasoconstriction triggering SDs after aSAH.^93^ For example, this hypothesis is supported by the fact that SD-induced spreading ischaemia leading to cerebral infarction started at a median p_ti_O_2_ of 12.5 (IQR: 9.2, 15.2) mmHg,^16^ which is already below the normal range.^94^ During spreading ischaemia, p_ti_O_2_ then fell further to 3.3 (2.4, 7.4) mmHg.^16^ Similarly, rCBF showed a downward trend even before the onset of SD-induced spreading ischaemia. Immediately before the onset of the spreading ischaemia leading to infarction, rCBF was 57 (53, 65) % compared to baseline and then dropped to 26 (16, 42) % during the spreading ischaemia.^16^ On the other hand, it argues against the hypothesis of vasoconstriction being responsible for SDs after aSAH that DSA-derived peripheral cerebral circulation time as a measure of microcirculatory resistance did not correlate with SD variables or DCI in patients.^95^ In addition, SD clusters after aSAH correlate strongly with clinical neurologic deficits, but there are cases of aSAH patients in whom SD clusters were not followed by delayed infarcts but only by reversible delayed vasogenic cortex oedema, reminiscent of MRI findings in familial hemiplegic migraine.^4^ In the present study, we cannot clarify the exact pathomechanisms by which SDs arise, but we found evidence that subarachnoid clots overlying the cortex are associated with SD variables, that SD variables are significantly associated with delayed infarcts, and that the SD component is a statistical mediator between subarachnoid blood and delayed infarcts. The fact that extravascular blood products and especially haemoglobin have complex degradation pathways^96^ that may vary from patient to patient and could have an important influence on the development of DCI and even beyond on patient outcome could not be considered in the present study for methodological reasons. Iron deposits, which are likely toxic and an end product of these degradation pathways, can still be detected in the cortex months after the initial haemorrhage, which may further worsen long-term patient outcomes.^61,97^

Analysis of angiographic vasospasm revealed that both the correlation of blood_inter_ and IVH with DSA_A2_ remained significant with strict Bonferroni correction. Furthermore, only two uncorrected significant results are expected by chance in 36 tests, but we observed such significances in a total of 8 correlations between blood and DSA variables (Table 3). In the correlation analyses between DSA variables and delayed infarcts, two correlations remained significant with Bonferroni correction. Without Bonferroni correction, we observed significances in eight correlations, while only one uncorrected significant result would be expected by chance. Overall, this supports an association between blood volume and DSA variables and another association between DSA variables and delayed infarcts. In the path analyses, the DSA component was a statistical mediator in a path from IVH to delayed infarcts. Possibly, blood products from the ventricles slowly move to the venous system via the glymphatic system, i.e. via para-arterial spaces. In this way, they could reach the arterial tunica media and induce a local and, via conduction mechanisms between myocytes, also more widespread vasospasm. A prominent role of IVH for angiographic vasospasm has been discussed previously, for example, in the context of angiographic vasospasm after rupture of arteriovenous malformations.^98,99^

SD and DSA variables did not correlate. Of 12 correlations between SD variables and peak mbfvs, 4 had uncorrected *P*-values <0.05, while <1 would have been expected by chance. However, none of these correlations remained significant after Bonferroni correction. Reduced perfusion from proximal vasospasm should favour SDs according to animal studies,^5,92^ but in agreement with previous clinical observations, we found no statistically significant evidence for this.^4,95^

Cerebral infarction is tissue death (necrosis) in which, in addition to SD/neuronal cytotoxic oedema, a lack of rCBF to the tissue, commonly referred to as ischaemia, occurred before the development of necrosis. As explained in the ‘Introduction’ section, cerebral ischaemia may occur primarily and trigger secondary SD/neuronal cytotoxic oedema with a delay of 1–5 min, such as after MCA occlusion,^16^ or SD/neuronal cytotoxic oedema may occur primarily, e.g. as a result of primary neuronal or astrocytic disruption or local inflammation, and trigger spreading ischaemia within seconds via the mechanism of the inverse haemodynamic response.^5,21^ The standard experimental protocol for causing spreading ischaemia is brain topical application of aCSF containing elevated [K^+^]_aCSF_ combined with either an NOS inhibitor or the NO scavenger haemoglobin.^21^ The original hypothesis in 1998 that spreading ischaemia might be a pathophysiological correlate of delayed infarcts after aSAH was based on the consideration that the release of blood products from the clot creates a microenvironment similar to that which experimentally leads to spreading ischaemia.^21^ Indeed, the phenomenology of spreading ischaemia later recorded in aSAH patients using subdural optoelectrode strips and oxygen sensors is not different from experimentally recorded spreading ischaemia in the animal model.^4,16,22^ In 2018, using neuromonitoring in combination with longitudinal neuroimaging, the entire sequence of infarct development after aSAH with SD-induced persistent activity depression, SD-induced spreading ischaemia and transition from clustered SDs to NUP was demonstrated in a small patient population where the recording devices were located directly in the area of newly developing infarcts.^16^ The concept that local factors at the cortex surface suffice to initiate the mechanism of spreading ischaemia^21^ is also consistent with observations in DISCHARGE-1 that 14.5% of patients with delayed infarcts had no angiographic vasospasm and 50% had only relatively mild angiographic vasospasm.^4^ The often extreme hyperaemia typically observed in aSAH patients immediately following severe spreading ischaemia also argues against sustained upstream restriction of rCBF as the principal cause of spreading ischaemia, as sustained upstream restriction of rCBF would not allow hyperaemia of such high amplitude to occur (compare figure 7 in Dreier *et al*.^22^ and figure 6A in Luckl *et al*.^16^). Nevertheless, experimentally, upstream reduction in rCBF further shifts the normal haemodynamic response to SD towards the inverse haemodynamic response.^70,100^ That is, proximal vasospasm should exacerbate spreading ischaemia, although this may not necessarily translate into a statistically significant change in SD count or depression periods, which, after all, are measured in 71% of patients with electrodes outside the ischaemic zone proper, i.e. outside the zone where spreading ischaemia occurs.^4^

## Conclusion

We found that SDs are a statistical mediator between subarachnoid blood and delayed infarcts. Our results suggest that especially the blood in sulci and fissures, which was usually not considered in previous analyses, plays a major role in the pathogenesis of delayed infarcts. Thus, delayed infarcts may depend on downstream rather than upstream mechanisms and on not only vascular but also important parenchymal factors. This may explain why robust antagonization of proximal vasospasm alone did not suffice to effectively prevent delayed infarcts.^101-103^ However, our results also support that angiographic vasospasm, SD and spreading ischaemia are not mutually exclusive pathomechanisms but complement each other, and we would therefore advocate that therapeutic combination approaches also be pursued further. A limitation of our study is the restricted spatial sampling with only six subdural electrodes. The majority of the electrodes were typically located over MCA territory. Accordingly, the correlation between SD variables and DCI_MCA_ was higher than the correlations between SD variables and DCI_ACA_ or DCI_PCA_. On the other hand, the fact that correlations between SD variables and DCI_ACA_ or DCI_PCA_ were also statistically significant illustrates once again that subdural neuromonitoring affords even remote detection of injury because SDs propagate widely from metabolically stressed zones.^72^ This is a particular advantage of ECoG over other neuromonitoring modalities, such as microdialysis and partial pressure of oxygen measurements, that measure only local conditions and may not detect clinically important changes developing elsewhere in the hemisphere. Remote diagnosis of new ischaemic zones is of particular relevance to patients with aSAH because the exact location of future developing pathology is usually unknown when the neurosurgeon implants neuromonitoring devices.

## Supplementary Material

fcad080_Supplementary_Data

## Abbreviations

ACA =: anterior cerebral artery
aCSF =: artificial cerebrospinal fluid
aSAH =: aneurysmal subarachnoid haemorrhage
blood_basal_ =: subarachnoid blood volume in the basal cisterns
blood_component_ =: first principal component of the blood volume variables
blood_convex_ =: subarachnoid blood volume on the cerebral convexity
blood_inter_ =: subarachnoid blood volume in the interhemispheric fissure
blood_Sylvian_ =: subarachnoid blood volume in the Sylvian fissure
CBF =: cerebral blood flow
COSBID =: Co-Operative Studies on Brain Injury Depolarisations
CTA =: computed tomography angiography
DC potential =: direct current (steady) potential
DCI =: delayed cerebral ischaemia
DCI_ACA_ =: delayed infarct volume in the territory of the anterior cerebral artery
DCI_deep_ =: delayed infarct volume below the cortex, including perforator infarcts, singular white matter infarcts without cortical involvement and anterior choroidal artery infarcts
DCI_MCA_ =: delayed infarct volume in the territory of the middle cerebral artery
DCI_PCA_ =: delayed infarct volume in the territory of the posterior cerebral artery
DCI_watershed_ =: delayed infarct volume in the territory of the cortical watershed zones
DISCHARGE-1 =: Depolarisations in ISCHaemia after subARachnoid haemorrhaGE-1
DSA =: digital subtraction angiography [A1, A2, M1, M2, P1, P2 = first and second segments of anterior cerebral artery (ACA), middle cerebral artery (MCA) and posterior cerebral artery (PCA) ipsilateral to the subdural electrodes]
DSA_component_ =: first principal component of the digital subtraction angiography variables to quantify the degree of vasospasm
ECI =: early cerebral ischaemia
ECI_ACA_ =: early infarct volume in the territory of the anterior cerebral artery
ECI_deep_ =: early infarct volume below the cortex including perforator infarcts, singular white matter infarcts without cortical involvement and anterior choroidal artery infarcts
ECI_MCA_ =: early infarct volume in the territory of the middle cerebral artery
ECI_PCA_ =: early infarct volume in the territory of the posterior cerebral artery
ECI_watershed_ =: early infarct volume in the territory of the cortical watershed zones
ECoG =: electrocorticography
eGOS =: extended Glasgow Outcome Scale
FLAIR =: fluid-attenuated inversion recovery
ICH =: intracerebral haemorrhage
Image_early_ =: post-interventional MRI or CT performed no later than Day 5
Image_late_ =: follow-up MRI or CT performed after the end of neuromonitoring around Day 14
IQR =: interquartile range
IVH =: intraventricular haemorrhage
[K^+^]_aCSF_ =: potassium concentration in the artificial cerebrospinal fluid
mbfv =: transcranial Doppler-sonography (TCD)-determined mean blood flow velocity
mbfv_ACA_ =: TCD-determined peak mean blood flow velocity of the anterior cerebral artery
mbfv_component_ =: first principal component of the TCD-determined peak mean blood flow velocity variables
mbfv_MCA_ =: TCD-determined peak mean blood flow velocity of the middle cerebral artery
mbfv_PCA_ =: TCD-determined peak mean blood flow velocity of the posterior cerebral artery
MCA =: middle cerebral artery
NO =: nitric oxide
NOS =: nitric oxide synthase
NUP =: negative ultraslow potential
pc =: path coefficients
PCA =: posterior cerebral artery
peak_clusSD-delayed_ =: peak number of clustered spreading depolarizations (SD) of a recording day during the delayed period between the early post-intervention neuroimage and the late neuroimage after completion of neuromonitoring (clustered SD = SD that occurred less than 1 h apart from the previous SD)
peak_isoSD-delayed_ =: peak number of isoelectric SDs of a recording day during the delayed period (isoelectric SD = SD in electrically inactive tissue)
peak_SD-delayed_ =: peak number of SDs of any type of a recording day during the delayed period
PTDDD_delayed_ =: peak value of a recording day for the total (cumulative) SD-induced depression durations during the delayed period
rCBF =: regional cerebral blood flow
SD =: spreading depolarization
TCD =: transcranial Doppler-sonography
TDDD =: total (cumulative) spreading depolarization-induced depression duration of a recording day
