# Supplementary material for: Spreading depolarization and angiographic spasm are separate mediators of delayed infarcts
Source: Brain Commun. 2023 Mar 22;5(2):fcad080. doi: 10.1093/braincomms/fcad080 (PMC10082345; doi:10.1093/braincomms/fcad080)
Supplement: fcad080_Supplementary_Data [file fcad080_supplementary_data.pdf]

Supplementary Material

Supplementary materials and methods

Digital subtraction angiography (DSA) grading score

VK determined vascular narrowing using a qualitative grading score (no vascular narrowing = 1, vascular narrowing by 11 - 33% = 2, vascular narrowing by 34 - 66% = 3, vascular narrowing > 67% = 4) for all DSAs performed between Days 5 and 17 [median Day 7 (IQR: 7 - 8)] as suggested previously.[1] In the present paper, only the ipsilateral version of the score was used[2] which included the first and second segments of middle cerebral artery (MCA), anterior cerebral artery (ACA) and posterior cerebral artery (PCA) and the distal part of the internal carotid artery (ICA) ipsilateral to the recording strip (n = 106). From these assessments, the ipsilateral DSA score was calculated (total score achieved divided by the number of vessel segments assessed).

In 66 patients, both an initial DSA on median Day 0 [interquartile range (IQR): 0-0] and a follow-up DSA on median Day 8 [IQR: 7-9] were available, so that a quantitative analysis of the angiographic vasospasm was possible to check the validity of the qualitative analysis against the quantitative analysis. As proposed previously,[1] for the purpose of the quantitative analysis, the absolute values of the diameters of the M1 segment of both MCAs, the A1 segment of both ACAs, the P1 segment of both PCAs, the distal parts of both ICAs, the basilar artery (BA) and both intradural vertebral arteries (VA, V4 segment) were measured in both DSAs. For each vessel segment, ratios in percent were calculated from its absolute value in relation to either the absolute value of the petrous segment of the ICA or the extradural vertebral artery (V3 segment). The V3 segment with the largest diameter was used as a reference for the BA. Subsequently, relative changes of the diameter ratios from the initial DSA to the follow-up DSA were calculated in percent. In addition to this quantitative analysis, another qualitative analysis was performed for the 66 patients according to the same scheme as above, identifying no, mild, moderate or severe vasospasm, but considering the same vascular segments as in the quantitative analysis (i.e. both M1, A1, P1, ICA and V4 segments as well as the BA). An important difference between the qualitative and quantitative score was that the quantitative score involved a punctual measurement at the narrowest point of the respective vessel segment,

whereas the qualitative score assessed the overall impression of the respective vessel segment.

We found that both bilateral quantitative and bilateral qualitative DSA score were significantly correlated with each other (Spearman correlation coefficient: 0.51, $P < 0.001$, n = 66) (**Supplementary Figure 1A**). However, we also correlated each of the two scores with the previously determined volume of bilateral supra- and infratentorial delayed ischaemic infarction[2] and found that the delayed infarct volume only correlated with the qualitative grading score (Spearman correlation coefficient: 0.40, $P < 0.001$, n = 66) but not with the quantitative grading score (Spearman correlation coefficient: 0.04, $P = 0.749$, n = 66) (**Supplementary Figure 1B**). Thus, while the statistical association between the qualitative DSA score and the volume of delayed infarcts could also be confirmed in this analysis of total delayed damage, the quantitative DSA score had no predictive value for the volume of delayed infarcts in this analysis.

Supplementary references

1. Weidauer S, Lanfermann H, Raabe A, Zanella F, Seifert V, Beck J. Impairment of cerebral perfusion and infarct patterns attributable to vasospasm after aneurysmal subarachnoid hemorrhage: a prospective MRI and DSA study. *Stroke*. Jun 2007;38(6):1831-6. doi:STROKEAHA.106.477976 [pii] 10.1161
2. Dreier JP, Winkler MKL, Major S, et al. Spreading depolarizations in ischaemia after subarachnoid haemorrhage, a diagnostic phase III study. *Brain*. May 24 2022;145(4):1264-1284. doi:10.1093/brain/awab457
3. Friedman JA, Goerss SJ, Meyer FB, et al. Volumetric quantification of Fisher Grade 3 aneurysmal subarachnoid hemorrhage: a novel method to predict symptomatic vasospasm on admission computerized tomography scans. *J Neurosurg*. Aug 2002;97(2):401-7. doi:10.3171/jns.2002.97.2.0401
4. Reilly C, Amidei C, Tolentino J, Jahromi BS, Macdonald RL. Clot volume and clearance rate as independent predictors of vasospasm after aneurysmal subarachnoid hemorrhage. *J Neurosurg*. Aug 2004;101(2):255-61. doi:10.3171/jns.2004.101.2.0255
5. Ko SB, Choi HA, Carpenter AM, et al. Quantitative analysis of hemorrhage volume for predicting delayed cerebral ischemia after subarachnoid hemorrhage. *Stroke*. Mar 2011;42(3):669-74. doi:10.1161/STROKEAHA.110.600775
6. Zijlstra IA, Gathier CS, Boers AM, et al. Association of Automatically Quantified Total Blood Volume after Aneurysmal Subarachnoid Hemorrhage with Delayed Cerebral Ischemia. *AJNR Am J Neuroradiol*. Sep 2016;37(9):1588-93. doi:10.3174/ajnr.A4771
7. van der Steen WE, Zijlstra IA, Verbaan D, et al. Association of Quantified Location-Specific Blood Volumes with Delayed Cerebral Ischemia after Aneurysmal Subarachnoid Hemorrhage. *AJNR Am J Neuroradiol*. Jun 2018;39(6):1059-1064. doi:10.3174/ajnr.A5626
8. van der Steen WE, Leemans EL, van den Berg R, et al. Radiological scales predicting delayed cerebral ischemia in subarachnoid hemorrhage: systematic review and meta-analysis. *Neuroradiology*. Mar 2019;61(3):247-256. doi:10.1007/s00234-019-02161-9

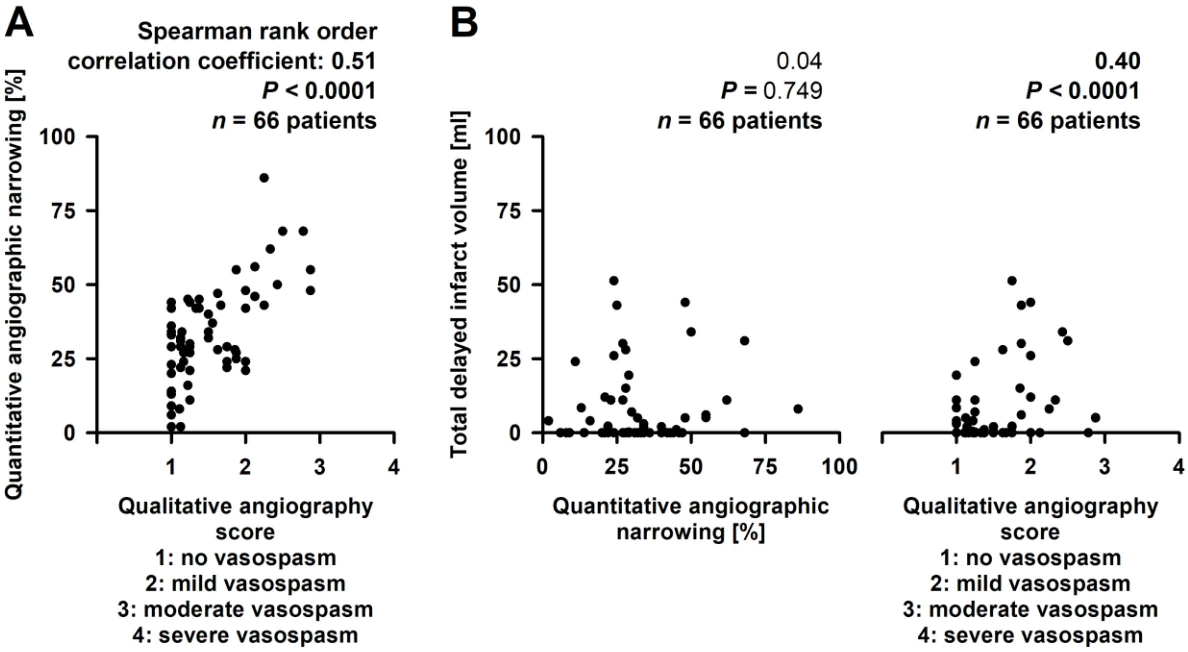

Supplementary figure 1 Quantitative versus qualitative digital subtraction angiography (DSA) score. (**A**) In 66 patients, both an initial DSA on median Day 0 [interquartile range (IQR): 0-0] and a follow-up DSA on median Day 8 [IQR: 7-9] were available, so that a quantitative analysis of the angiographic vasospasm was possible to check the validity of the qualitative analysis against the quantitative analysis. For these analyses, both the quantitative score and the qualitative score were based on the M1 segment of both middle cerebral arteries (MCA), the A1 segment of both anterior cerebral arteries (ACA), the P1 segment of both posterior cerebral arteries (PCA), the distal parts of both internal carotid arteries (ICA), the basilar artery (BA) and both intradural vertebral arteries (VA, V4 segment). (**A**) Correlation between quantitative and qualitative score. (**B**) For the comparison of the quantitative and qualitative DSA score with the volume of delayed infarcts, the total volume of delayed infarcts in both the ipsilateral and contralateral cerebral hemispheres and in the infratentorial brain including brainstem and cerebellum was considered. All delayed infarct volumes have previously been calculated by VH in connection with the DISCHARGE-1 study.[2] VK previously determined both the quantitative and qualitative DSA score blinded to the neuroimaging results and other variables in connection with the DISCHARGE-1 study.[2] In contrast to the qualitative DSA score, the quantitative DSA score did not correlate with the total delayed infarct volume. This could be due to the fact that, in contrast to the qualitative score, the length of spastic segments was not taken into account in the quantitative score.

Supplementary Table 1 Spearman correlations between potential mediators

Statistical Analysis	Variable	Spearman coefficient	*P* value	Number of patients
**Association analysis with $PTDDD_{delayed}$**	**DSA_{A1}**	0.05	0.645	103
	**DSA_{A2}**	0.00	0.986	104
	**DSA_{M1}**	0.14	0.169	105
	**DSA_{M2}**	0.06	0.538	105
	**DSA_{P1}**	0.02	0.859	91
	**DSA_{P2}**	-0.02	0.832	90
	**$mbfv_{ACA}$**	0.12	0.222	112
	**$mbfv_{MCA}$**	0.08	0.360	128
	**$mbfv_{PCA}$**	**0.25**	**0.012**	98
**Association analysis with $peak_{SD\text{-}delayed}$**	**DSA_{A1}**	0.05	0.633	103
	**DSA_{A2}**	0.00	0.988	104
	**DSA_{M1}**	0.10	0.326	105
	**DSA_{M2}**	0.03	0.788	105
	**DSA_{P1}**	0.05	0.656	91
	**DSA_{P2}**	0.01	0.942	90
	**$mbfv_{ACA}$**	**0.21**	**0.027**	112
	**$mbfv_{MCA}$**	0.11	0.232	128
	**$mbfv_{PCA}$**	**0.27**	**0.007**	98
**Association analysis with $peak_{isoSD\text{-}delayed}$**	**DSA_{A1}**	-0.04	0.696	103
	**DSA_{A2}**	-0.06	0.520	104
	**DSA_{M1}**	0.09	0.358	105
	**DSA_{M2}**	0.00	0.995	105
	**DSA_{P1}**	0.03	0.794	91
	**DSA_{P2}**	-0.06	0.606	90
	**$mbfv_{ACA}$**	0.18	0.055	112
	**$mbfv_{MCA}$**	0.09	0.319	128
	**$mbfv_{PCA}$**	0.10	0.345	98
**Association analysis with $peak_{clusSD\text{-}delayed}$**	**DSA_{A1}**	0.05	0.650	103
	**DSA_{A2}**	-0.04	0.692	104
	**DSA_{M1}**	0.16	0.105	105
	**DSA_{M2}**	0.00	0.998	105
	**DSA_{P1}**	0.08	0.454	91
	**DSA_{P2}**	-0.02	0.825	90
	**$mbfv_{ACA}$**	0.18	0.060	112
	**$mbfv_{MCA}$**	0.12	0.163	128
	**$mbfv_{PCA}$**	**0.21**	**0.039**	98
**Association analysis with $mbfv_{ACA}$**	**DSA_{A1}**	0.14	0.206	84
	**DSA_{A2}**	0.01	0.911	85
	**DSA_{M1}**	**0.28**	**0.011**	86
	**DSA_{M2}**	**0.22**	**0.041**	86
	**DSA_{P1}**	0.16	0.160	77
	**DSA_{P2}**	0.05	0.658	76
**Association analysis with $mbfv_{MCA}$**	**DSA_{A1}**	0.17	0.094	95
	**DSA_{A2}**	0.10	0.355	96
	**DSA_{M1}**	**0.37**	**< 0.001**	97
	**DSA_{M2}**	**0.25**	**0.014**	97
	**DSA_{P1}**	0.18	0.101	84
	**DSA_{P2}**	0.20	0.064	83
**Association analysis with $mbfv_{PCA}$**	**DSA_{A1}**	0.02	0.886	83
	**DSA_{A2}**	0.08	0.489	84
	**DSA_{M1}**	0.19	0.090	85
	**DSA_{M2}**	0.14	0.188	85
	**DSA_{P1}**	0.15	0.194	75
	**DSA_{P2}**	0.16	0.173	75

Legend Supplementary Table 1: All given data only refer to the hemisphere ipsilateral to the subdural electrodes. DSA = digital subtraction angiography [A1, A2, M1, M2, P1, P2 = first and second segments of anterior cerebral artery (ACA), middle cerebral artery (MCA) and posterior cerebral artery (PCA) ipsilateral to the subdural electrodes]; $mbfv_{ACA}$ = transcranial Doppler-sonography (TCD)-determined peak mean blood flow velocity of ACA; $mbfv_{MCA}$ = TCD-determined peak mean blood flow velocity of MCA; $mbfv_{PCA}$ = TCD-determined peak mean blood flow velocity of PCA; $peak_{clusSD\text{-}delayed}$ = peak number of clustered spreading depolarisations (SD) of a recording day during the delayed period between the early post-intervention neuroimage and the late neuroimage after completion of neuromonitoring (clustered SD = SD that occurred less than one hour apart from the previous SD); $peak_{isoSD\text{-}delayed}$ = peak number of isoelectric SDs of a recording day during the delayed period (isoelectric SD = SD in electrically inactive tissue); $peak_{SD\text{-}delayed}$ = peak number of SDs of any type of a recording day during the delayed period; $PTDDD_{delayed}$ = peak value of a recording day for the total (cumulative) SD-induced depression durations during the delayed period. Strictly speaking, TCD measures the mbfvs in the A1 segment of the ACA, M1 segment of the MCA and P2 segment of the PCA.

Supplementary Table 2 Summary of studies related to volumetric quantification of blood on the initial CT scan and delayed cerebral ischaemia (DCI) in patients with subarachnoid haemorrhage

Author (Date)	*n*	DCI [%]	Blood quantification	Endpoint	Main results
**Friedman et al. (2002)[3]**	40	70	Cisternal, intraventricular, intracerebral	DND	Median cisternal blood volume was 30.5ml in the severe DND group compared to 12.4ml in the moderate DND group and 10.3ml in the non-DND group ($P < 0.001$). Intraventricular and intracerebral blood were not significant.
**Reilly et al. (2004)[4]**	75	35	Cisternal	Symptomatic vasospasm	Multivariate logistic regression: OR 1.08 (1.02-1.15, $p = 0.011$) per ml increase in cisternal blood volume
**Ko et al. (2011)[5]**	160	25	Cisternal plus intraventricular blood	DCI	Cisternal plus intraventricular haemorrhage were classified into quartiles. Compared to the lowest quartile, the adjusted OR increased in a dose-related manner for each quartile (2.6, 4.5, 6.1, *P* for trend = 0.01).
**Zijlstra et al. (2016)[6]**	333	20	Total blood volume	DCI	Multivariate logistic regression: adjusted OR 1.02 (1.01-1.03) per ml increase in total blood volume.
**Van der Steen et al. (2018)[7]**	282	22	Cisternal, intraventricular, intracerebral, subdural	DCI	Multivariate logistic regression: adjusted OR 1.02 (1.01-1.04) per ml increase in cisternal blood volume. Intraventricular, intracerebral and subdural blood were not significant.
**Van der Steen et al. (2019)[8]**	369	31	Total blood volume	DCI	Only total blood volume was included in the final logistic regression model out of 10 potential predictors. $R^2 = 6\%$, c-statistic = 0.64.

Legend Supplementary Table 2: DCI = delayed cerebral ischaemia, DND = delayed neurological deficit, *n* = number of patients, OR = odds ratio.
